# Genetic Characterization of Continually Evolving Highly Pathogenic H5N6 Influenza Viruses in China, 2012–2016

**DOI:** 10.3389/fmicb.2017.00260

**Published:** 2017-02-28

**Authors:** Meng Li, Na Zhao, Jing Luo, Yuan Li, Lin Chen, Jiajun Ma, Lin Zhao, Guohui Yuan, Chengmin Wang, Yutian Wang, Yanhua Liu, Hongxuan He

**Affiliations:** ^1^National Research Center for Wildlife-Borne Diseases, Institute of Zoology, Chinese Academy of SciencesBeijing, China; ^2^College of Life Science, University of Chinese Academy of SciencesBeijing, China; ^3^Department of Animal Science, Hebei Normal University of Science and TechnologyQinghuangdao, China; ^4^Department of Microbiology, Beijing General Station of Animal HusbandryBeijing, China

**Keywords:** highly pathogenic avian influenza, H5N6, *Pavo cristatus*, phylogenetic analysis, molecular characterization, genetic reassortment

## Abstract

H5N6 is a highly pathogenic avian influenza (HPAI) and a zoonotic disease that causes recurring endemics in East Asia. At least 155 H5N6 outbreaks, including 15 human infections, have been reported in China. These repeated outbreaks have increased concern that the H5N6 virus may cross over to humans and cause a pandemic. In February, 2016, peafowls in a breeding farm exhibited a highly contagious disease. Post-mortem examinations, including RT-PCR, and virus isolation, confirmed that the highly pathogenic H5N6 influenza virus was the causative agent, and the strain was named A/*Pavo Cristatus*/Jiangxi/JA1/2016. In animal experiments, it exhibited high pathogenicity in chickens and an estimated median lethal dose in mice of ~10^4.3^ TCID_50_. A phylogenetic analysis showed that JA1/2016 was clustered in H5 clade 2.3.4.4. FG594-like H5N6 virus from Guangdong Province was the probable predecessor of JA1/2016, and the estimated divergence time was June 2014. Furthermore, we found that H5N6 influenza viruses can be classified into the two following groups: Group 1 and Group 2. Group 2 influenza viruses have not been detected since the end of 2014, whereas Group 1 influenza viruses have continually evolved and reassorted with the “gene pool” circulating in south China, resulting in the rise of novel subtypes of this influenza virus. An increase in the number of its identified hosts, the expanding range of its distribution, and the continual evolution of H5N6 AIVs enhance the risk that an H5N6 virus may spread to other continents and cause a pandemic.

## Introduction

Highly pathogenic avian influenza (HPAI) is one of the most concerning infectious diseases in the world (Taubenberger and Morens, 2008). Highly pathogenic avian influenza virus (HPAIV) infections usually cause a highly contagious systemic disease with significant morbidity and mortality in susceptible populations, resulting in severe economic losses (Bevins et al., 2016a). Low pathogenic avian influenza (LPAI) viruses usually trigger milder and primarily respiratory disease in birds. In most cases, LPAI viral infections have little impact on the poultry industry, but the threat they pose to public health cannot be ignored. First, LPAI viruses occasionally induce relatively high mortality in chicks (Alexander, 2000). Second, some LPAI viruses have resulted in lethal infections in humans. These include the H7N9 virus, which causes endemic in China (Belser et al., 2016b; Zhu et al., 2016). LPAI and HPAI viruses co-circulate around the world, comprising a viral “gene pool.” As a result of the accumulation of site mutations, genetic reassortment, and genetic recombination, novel AIVs arise quickly and frequently, causing repeated outbreaks in birds and sporadic cases in humans (Durrant et al., 2016). Although not yet a pandemic, these recurring AIV outbreaks pose a persistent potential pandemic threat throughout the world.

Since 2009, the worldwide dissemination of H5 clade 2.3.4.4 HPAI viruses has become a serious threat to the poultry industry in China (Smith et al., 2015). Outbreaks of viruses in this clade have included the H5N1, H5N2, H5N6, and H5N8 subtypes (Claes et al., 2016). In 2014, the first outbreak of the HPAI H5N6 virus was confirmed in a poultry farm in Nanchong City, Sichuan Province, China (FAO). Approximately 155 outbreaks have been reported in 18 provinces (and/or autonomous regions) across China, including 15 cases of human infections (FAO, Supplementary Figure 1). Additionally, at least 20 outbreaks have also been reported in Laos and Vietnam (FAO). Importantly, H5N6 is the only subtype among the clade 2.3.4.4 viruses that has caused human infections (Shen et al., 2015; Yang et al., 2015; Pan et al., 2016). The recurrence of these outbreaks has increased concern that H5N6 viruses may cross over to mammals. Moreover, the geographic distributions of H5N6 viruses and their hosts have considerably expanded (Supplementary Figure 1). There is no evidence that H5N6 influenza viruses have been transmitted in another continent, but HPAI H5N6 viruses have been detected in migratory birds, and this increases the risk that these viruses could spread to other countries or continents (Bi et al., 2016).

Here, we investigated the virologic and phylogenetic characteristics of the HPAI H5N6 virus in the *Pavo Cristatus* breeding farm in Jiangxi Province, China. Furthermore, we analyzed the co-circulation of H5N6 AIVs within the “viral gene pool” that is circulating in Asia, Europe, and North America. The results of this study reveal several characteristics of H5N6 virus evolution.

## Materials and methods

### Ethics statement

These animal studies were performed in strict accordance with the Guidelines for the Care and Use of Animals in Research, which are issued by the Institute of Zoology, Chinese Academy of Sciences. This study was evaluated and approved by the Animal Ethics Committee of the Institute of Zoology, Chinese Academy of Sciences. All experiments were conducted in a Biosafety Level 3 (BSL-3) facility.

### Sample collection

Swab samples, tissue samples, and environmental samples were collected from the breeding farm. More specifically, oropharyngeal, and cloacal swabs were collected from affected peafowls. Swab samples were placed in 10% w/v PBS buffer containing antibiotics (penicillin and streptomycin, 2,000 IU) and then ground and centrifuged to collect supernatants for RNA extraction and virus isolation. Tissue samples (i.e., the brain, lungs, heart, liver, spleen, intestines, and kidneys) were collected under aseptic conditions and then stored and transported on ice. Environmental samples, including water, food, or fecal samples, were collected and stored as swab samples.

### Virus isolation and sequencing

The inoculum for each sample was propagated in 11-day-old SPF chicken eggs (Vital River). Allantoic fluids were harvested from eggs that had died within 72 h after inoculation. The influenza virus was isolated, sequenced, and named A/*Pavo Cristatus*/Jiangxi/JA1/2016 (H5N6). The following primers were used for genome sequencing, as previously reported (Kreibich et al., 2009):

Uni12: 5-AGCRAAAGCAGG-3′;
UnIA-F^a^: 5′-GAAGTTGGGGGGGAGCAAAAGCAGG-3′ and
UnIA-R^b^: 5′-CCGCCGGGTTATTAGTAGAAACAAGG-3′;
PB2-882F^c^: 5′-TGGTTGACATCCTTAGACAAAAC-3′ and
PB2-1568R^d^: 5′-CCCTGTGTTTCACTAACCTC-3′;
PB1-1178F^c^: 5′- CAAGAGAGAAAATCGAGAAAATAAG-3′ and
PB1-1354R^d^: 5′-AGGATTGGAGTCCGTCCCACCAATATGT-3′; and
PA-1243F^c^: 5′-ATCCAGAATGAATTCAACAA-3′ and
PA-1423R^d^: 5′-TATTTATGTACACTCCCTTCATTAT-3′.
^a^ and ^b^: primers used to amplify HA, NA, NP, M, and NS gene segments;
^a^ and^d^: primers used to separately amplify the upper section of the PB2, PB1, and PA genes;
^b^ and^c^: primers used to separately acquire the second half of the PB2, PB1, and PA genes.

All PCR products were cloned into the pEASY-Blunt vector (Transgen) and sequenced (BGI). The results were assembled and submitted to the NCBI database.

### Phylogenetic analysis

The phylogenetic analyses were performed using the listed gene sequences, which were acquired during a NCBI BLAST analysis. H5- and N6-subtype influenza virus sequences that were deposited from 2012 to 2016 were downloaded from the NCBI and/or the GISAID database. The reference sequences used for the H5-subtype influenza virus classifications were chosen based on guidance from OIE. All of the segmented sequence datasets were aligned using ClustalW 1.6. The alignment lengths for each gene segment dataset were as follows: PB2, 2,280 nt; PB1, 2,271 nt; PA, 2,151 nt; HA, 1692 nt; NP, 1482 nt; NA, 1,413 nt; M, 981 nt; and NS, 822 nt.

Preliminary analyses were performed to estimate the best-fit nucleotide substitution model using jModeltest2 (Darriba et al., 2012). To estimate divergence times and rates of nucleotide substitutions, we used an uncorrelated relax-clock Bayesian Markov chain Monte Carlo method in BEAST v1.8.4 (Drummond et al., 2012). To determine which were the best-fit phylodynamic models, we performed different combinations of relaxed-clock models (i.e., exponential and log-normal models) and branch rate models (i.e., constant, exponential, expansion, and Bayersian SkyGrid models). The resultant ^*^log files were imported into Tracer (v1.6) and then analyzed using model comparison analyses (AICM analysis; Drummond and Bouckaert, 2015). The best fit substitution models, relaxed-clock models, and tree models are listed in Supplementary Tables S1, S2. For each analysis, MCMC chains were run for 50 million iterations and sampled every 5 1000 iterations after a 10% burn-in to ensure an effective sample size was obtained for all posterior parameters, including the posterior, prior, likelihood, and meanRate (ESS > 200). For Clade 2.3.4.4 subtype H5 AIVs and N6 subtype AIVs analyses, the MCMC chains were run for 400 million iterations. Maximum clade credibility (MCC) trees were obtained from the MCMC tree samples using TreeAnnotator v1.8.4 (Drummond et al., 2012). The resulting trees were edited and illustrated using FigTree (v1.4.2; Morariu et al., 2009).

### Animals experiment

To test the pathogenicity of the JA1/2016 virus, groups of five 6- to 8-week-old BALB/c mice (Vital River Laboratory, Beijing, China) were anesthetized using CO_2_ and then intranasally (i.n.) inoculated with 0.1 ml of serial 10-fold dilutions of infectious virus in PBS. The estimated median lethal dose was calculated and expressed as a TCID_50_ (50% tissue culture infectious dose) value. We inoculated 6-week-old chickens (*n* = 10) (Merial Vital laboratory, Beijing, China) intravenously with 0.1 ml of diluted allantoic fluid (hemagglutinin unit: 32). All of the chickens were killed by the infection within 24 h after inoculation. All of these experiments were conducted in a biosafety level 3 (BSL-3) facility.

## Results

### *Pavo cristatus* were killed by highly pathogenic avian influenza virus

**Figure 1:**
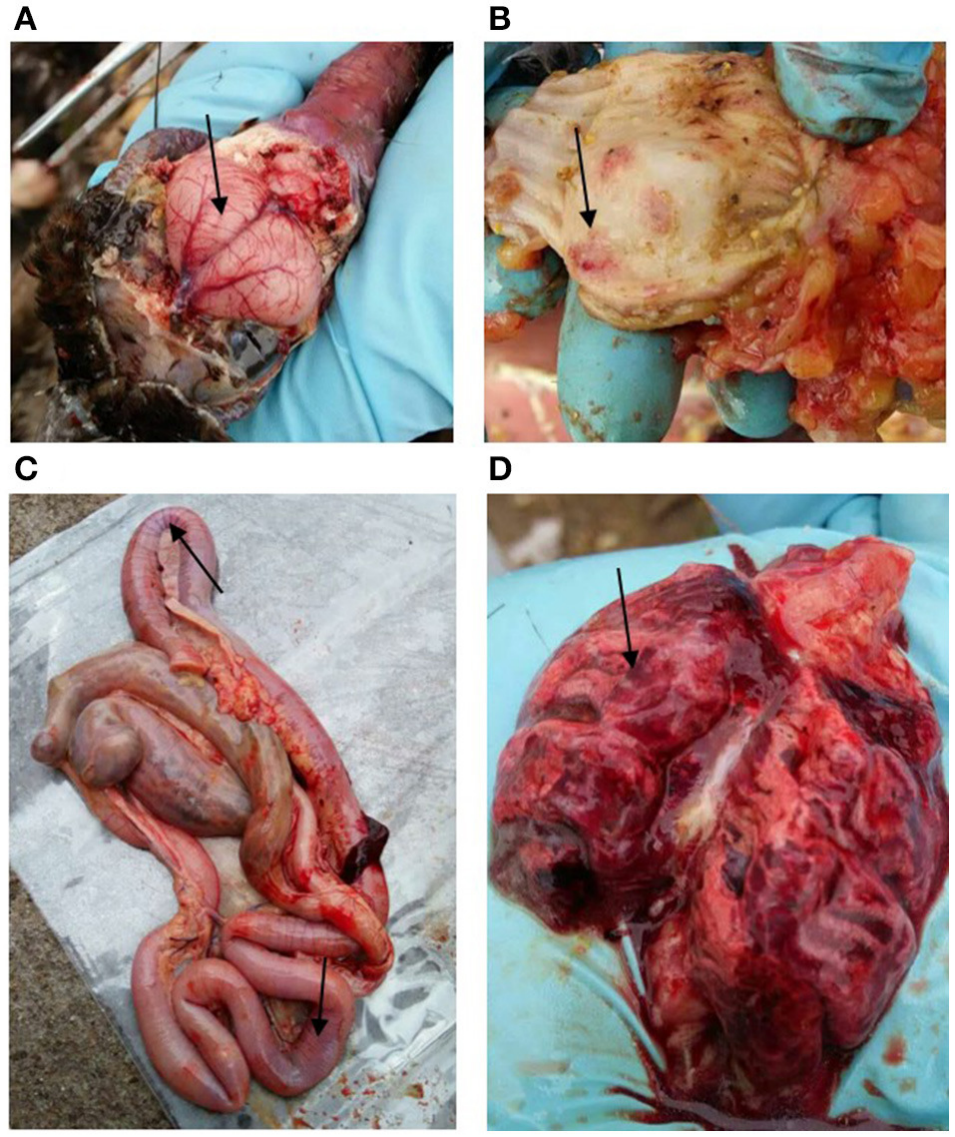
**Gross lesions in dead peafowls. (A)** Hyperemia and hemorrhaging were observed in the brain. In addition, severe hemorrhaging (black arrows) was observed in the glandular stomach **(B)**, intestine **(C)**, and lung **(D)**.

On February 20th, 2016, a few captive peafowls (*Pavo Cristatus*) were found dead in a breeding farm in Jiangxi Province, China. During the next week, more than 98% (770/787) of the farm peafowls were affected. All of the infected birds died within 24 h of the onset of clinical manifestations. Typical clinical signs, including neural system disorder and diarrhea, were observed, and these were consistent with the pathology observed in bar-headed geese that were infected with the HPAI H5N1 virus (Liu et al., 2005). Gross lesions, including those caused by gastrointestinal tract hemorrhaging, severe pneumonia, and encephalitis, were noted (Figure 1). All lesions were confirmed using histopathological analyses. Post-mortem examinations, RT-PCR detection, and virus isolation confirmed that the H5N6 influenza virus was the causative agent, and the strain was designated A/*Pavo Cristatus*/Jiangxi/JA1/2016 (abbreviated JA1/2016). No other subtype of influenza virus was detected.

### Genesis analysis of the novel H5N6 influenza virus

To understand the origin of JA1/2016, we performed blast analyses using the NCBI database. Our results demonstrated that JA1/2016 was most closely related to H5N6 AIVs that were previously isolated from Guangdong Province, China (Table 1). More phylogenetic information was estimated using MCC trees generated by BEAST (v1.8.4) software.

**Table 1:** **Gene segments of JA1/2016 demonstrating that it is most closely related to the H5N6 viruses that were isolated from Guangdong Province, China**.

**Gene**	**Virus strains**	**Identity (%)**
HA	A/chicken/Shenzhen/2269/2013 (H5N6)	98.3
	A/environment/Guangdong/QY197/2014 (H5N6)	
	A/chicken/Dongguan/2690/2013 (H5N6)	
	A/chicken/Zhejiang/727155/2014 (H5N6)	
NA	A/Chicken/Guangdong/FG594/2015 (H5N6)	99.3
	A/great_egret/Hong_Kong/00032/2016 (H5N6)	
PB2	A/duck/Vietnam/LBM760/2014 (H5N6)	99.3
	A/muscovy duck/Vietnam/LBM757/2014 (H5N6)	99.3
PB1	A/environment/Guangdong/GZ670/2015 (H5N6)	99.1
	A/Chicken/Guangdong/FG594/2015 (H5N6)	98.9
PA	A/Chicken/Guangdong/FG594/2015 (H5N6)	99.2
	A/environment/Guangdong/ZS558/2015 (H5N6)	99.1
NP	A/Chicken/Guangdong/FG594/2015 (H5N6)	99.3
	A/environment/Guangdong/GZ693/2015 (H5N6)	99.3
M	A/feline/Guangdong/1/2015 (H5N6)	99.6
	A/feline/Guangdong/2/2015 (H5N6)	99.6
NS	A/feline/Guangdong/1/2015 (H5N6)	99.2
	A/feline/Guangdong/2/2015(H5N6)	99.2

**Figure 2:**
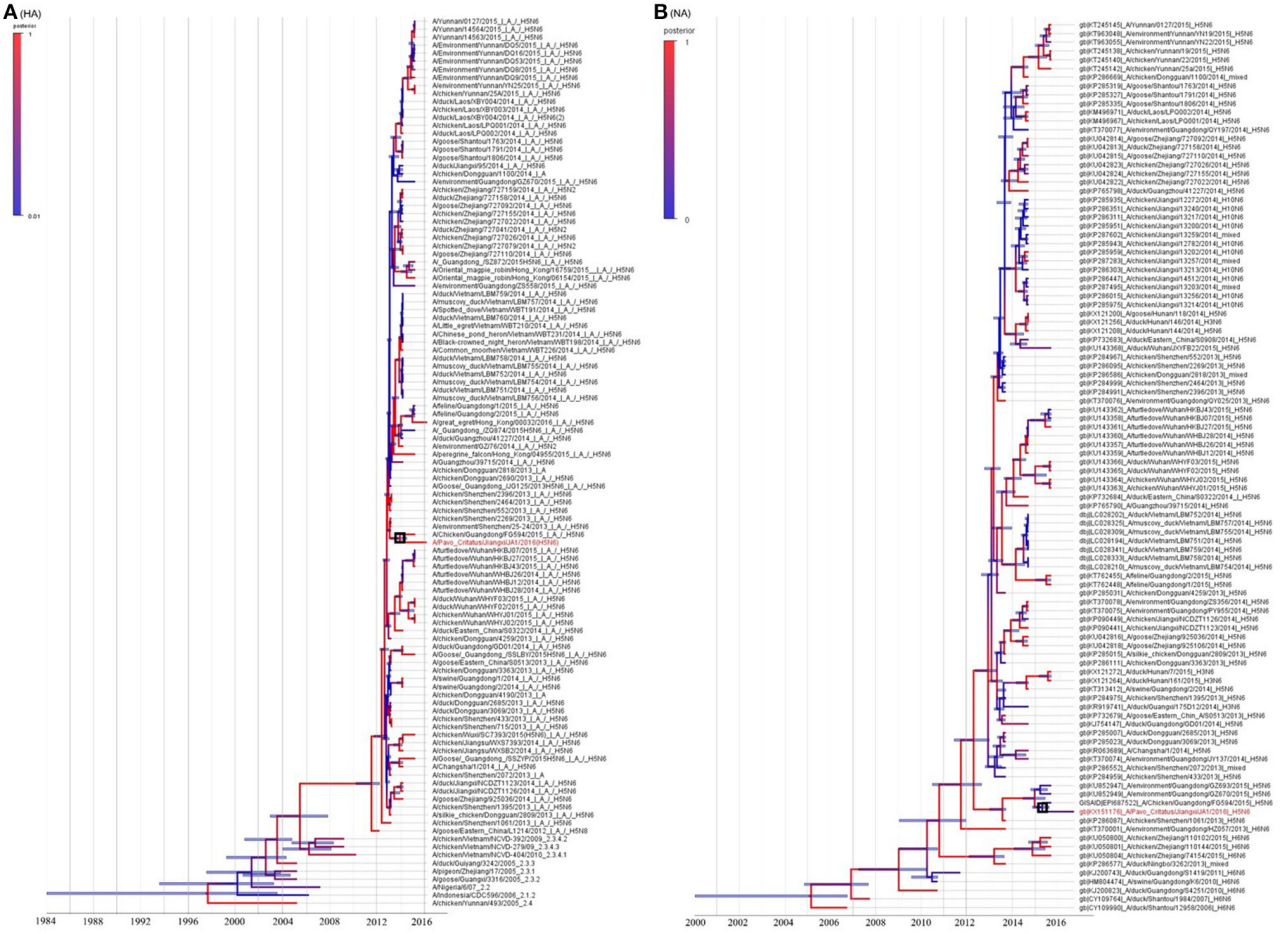
**Genetic analysis of JA1/2016**. The phylogenetic trees were constructed using gene sequences identified in NCBI or GISAID Blast analyses. Panels **(A,B)** represent the HA and NA genes, respectively. JA1/2016 is marked in red. The trees were built using BEAST (v1.8.4) and illustrated using FigTree (v1.4.2).

The resulting MCC trees suggested that the JA1/2016 hemagglutinin (HA) gene was clustered into the H5 clade 2.3.4.4 (Figure 2A), whereas its neuraminidase (NA) gene was derived from the Eurasian lineage (Figure 2B). The evolutionary rates (i.e., substitutions/site/year) of the eight gene segments of these H5N6 AIVs were estimated by Bayesian analysis (Table 2). Among the eight gene segments of H5N6 viruses, HA and NA genes had evolved slightly faster than the other analyzed gene segments, whereas the M gene evolved the slowest relative to the other analyzed segments. *Ne* estimation (i.e., number of genes effectively giving rise to the next generation) of HA and NA genes indicated an increase of the effective population size since 2013 (Supplementary Figure 3).

**Table 2:** **Estimated evolutionary rates of each gene segments of H5N6 viruses**.

**Genes**	**Substitution rate and 95% HPD (10^−3^ substitution/site/year)**		
	**Mean**	**Lower**	**Upper**
PB2	3.35	2.84	4.23
PB1	3.87	3.01	4.75
PA	2.92	2.20	3.68
HA	4.38	2.94	5.78
NP	2.91	2.01	3.90
NA	4.22	3.14	5.32
M	2.02	1.15	2.59
NS	2.95	2.23	3.65

**Figure 3:**
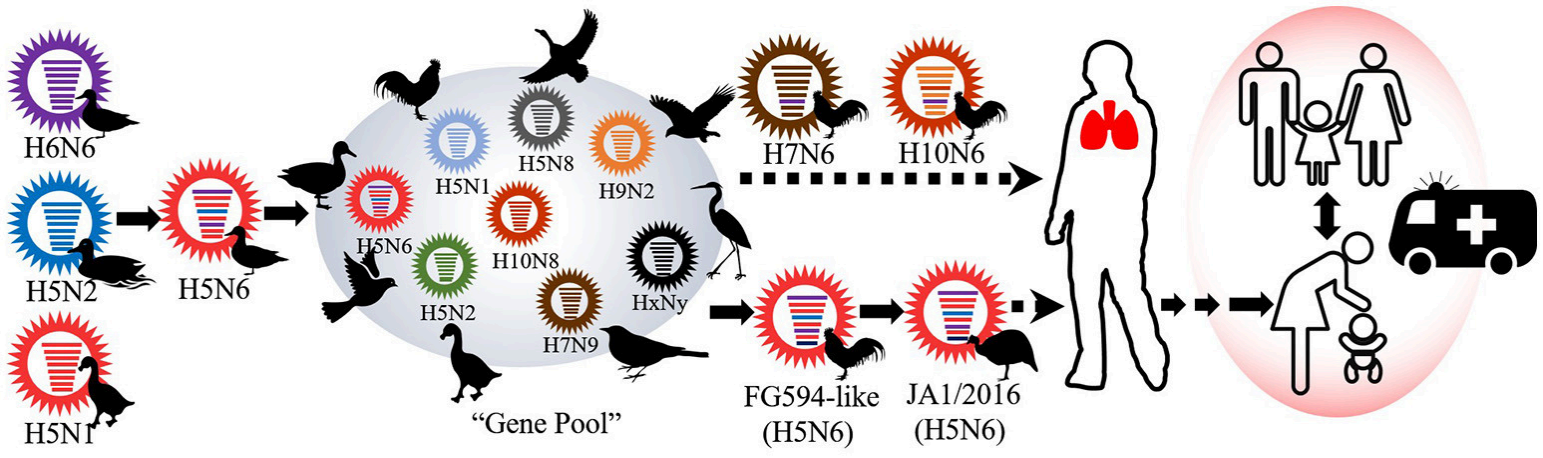
**The possible evolutionary history of the JA1/2016 virus**. The viral gene pool circulating in south and east China has played a pivotal role in the genesis of JA1/2016. However, the virus must be further adapted to efficiently infect humans.

Based on the MCC trees, we noted that the H5N6 subtype AIVs represented a triple-reassorted influenza virus that consisted of the H5N1, H5N2, and H6N6 influenza viruses, which is consistent with previous studies (Wong, 2015). The MCC trees derived using eight gene segments revealed that JA1/2016 shared the highest homology with A/Chicken/Guangdong/FG594/2015 (H5N6) and the following three genetically related H5N6 viruses: A/environment/Guangdong/GZ693/2015, A/environment/Guangdong/ZS558/2016, and A/Chicken/Guangdong/GZ670/2015 (Supplementary Figures 2A–F; Yuan et al., 2016). These results prompted us to consider that the FG594-like H5N6 virus from Guangdong Province was the probable predecessor of JA1/2016, and the estimated divergence time was June 2014 (Figure 3).

### Molecular characteristics of JA1/2016

To further understand the potential of JA1/2016 to cause a pandemic in mammals, we analyzed its molecular characteristics. The results suggested that JA1/2016 possessed multiple basic amino acids (-PLRERRRKR-) at an HA cleavage site, indicating that it is highly pathogenic to chickens (Supplementary Table S1; Webster and Rott, 1987). The Q222L and G224S substitutions in HA (H5 numbering system) suggested tropism in the avian-type receptor (SA-alpha-2,3 Gal; Supplementary Table S1; Matrosovich et al., 2000). In the NA protein, an 11-amino-acid deletion was found in the stalk region (residues 59–69). This deletion could influence the replication, virulence, and host range of the virus (Matrosovich et al., 1999). Residues in PA (L672), PB1 (H99, I368), and PB2 (T271, Q591, E627, D701) indicated that the virus was capable of air-borne transmission but showed low adaption to mammals (Steel et al., 2009; Bussey et al., 2010; Yamada et al., 2010; Herfst et al., 2012; Zhong et al., 2014). No drug resistance-associated mutations were found in the M2 protein (Supplementary Table S1). However, some key molecular features that have been associated with increased virulence in mammals were found in JA1/2016 (Supplementary Table S1). For example, a truncated PB1-F2 was found in JA1/2016, and this could increase its likelihood of adapting to mammals (Neumann et al., 2009). The NS1 protein has a 5-aa deletion at position 80–84 that also increases the virulence of the virus in mice (Zhu et al., 2008). Hence, from a molecular analysis point of view, several key steps are required for JA1/2016 to efficiently infect mammals.

### Pathogenicity of JA1/2016 in chickens and mice

To assess its virulence, mice and chickens were artificially infected with JA1/2016. The intravenous pathogenicity index (IVPI) of JA1/2016 was 3.0. All of the experimentally infected chickens died within 24 h of infection. Typical clinical signs were observed, including facial swelling, diarrhea, fluffed feathers, and huddling behaviors. BALB/c mice were also used to estimate the virus' pathogenicity in mammals, including humans. The results showed that the estimated median lethal dose for the HPAI H5N6 virus was ~10^4.3^ TCID_50_. Infection was restricted to the lungs, and no viral RNA or particles were detected in the brain or intestines.

### Genetic characteristics of H5 clade 2.3.4.4 subtype AIV isolates collected from 2012 to 2016

Genetic reassortment and recombination are the major sources of novel emerging AIVs. A “viral gene pool,” which is established by co-circulation among a diversity of AIVs, is persistent in Asia (Chen et al., 2016). This “gene pool” shares surface and/or internal viral genes with H5N6 isolates that produce novel variant AIVs. To explore the interface between H5N6 HPAIVs and the “viral gene pool,” we performed a phylogenetic analysis using H5- and N6-subtype isolates that were collected in Asia, Europe and North America from 2012 to 2016. The MCC trees revealed some of the characteristics of Clade 2.3.4.4 AIV evolution.

**Figure 4:**
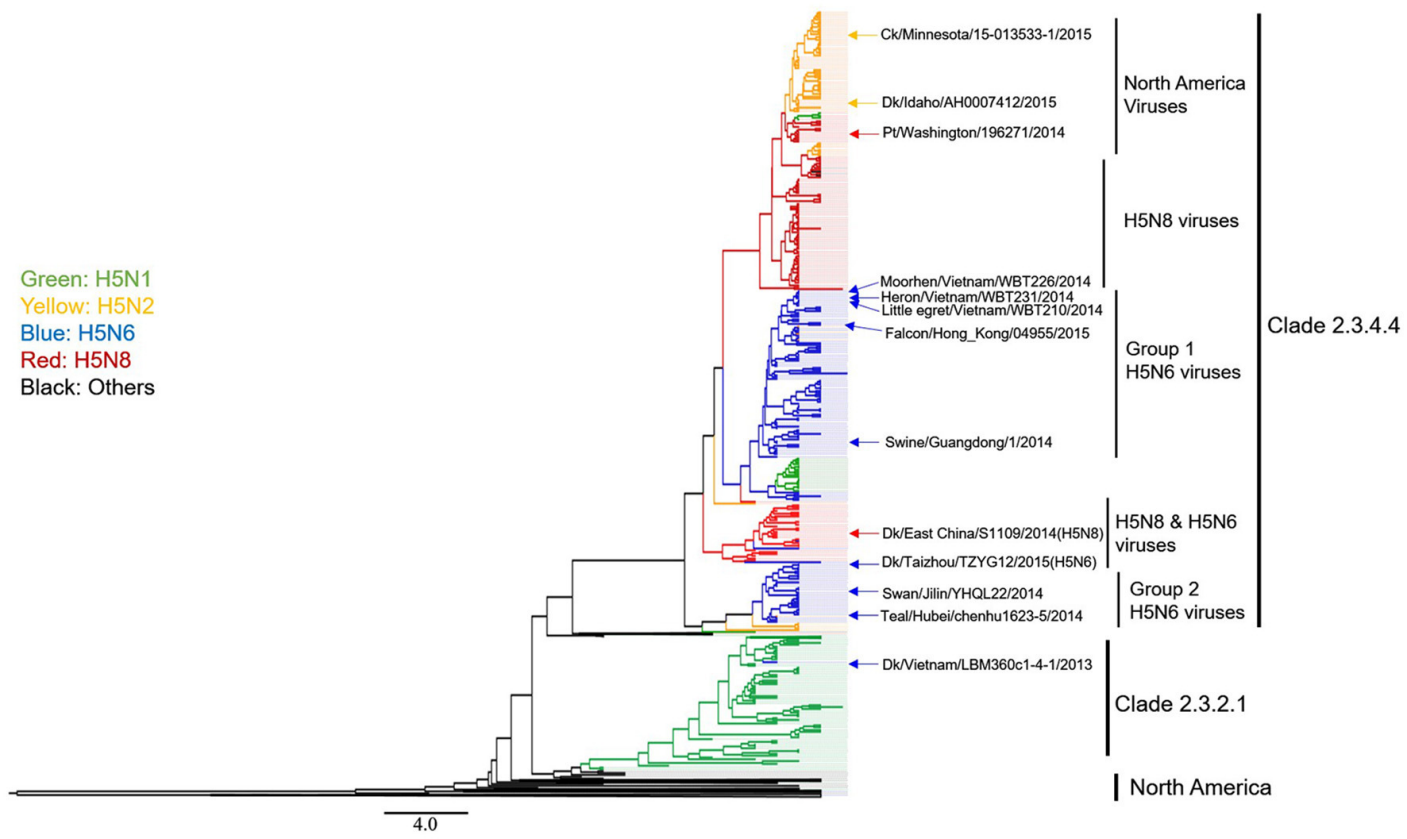
**Phylogenetic analysis of H5 subtype influenza viruses identified in 2012–2016 in Asia, Europe, and North America**. The MCC trees were built using BEAST (v1.8.4) and illustrated using FigTree (v1.4.2).

First, we found that H5 Clade 2.3.4.4 AIVs comprised the following several subtype AIVs: H5N1, H5N2, H5N6, H5N8, and H5N3. H5N6 and H5N8 AIVs are the most common members of the clade 2.3.4.4 viruses (Figure 4). Based on a phylogenetic tree, we found that H5N6 subtype AIVs had already donated their HA gene to the H5N1, H5N2, and H5N8 subtype AIVs, causing these AIVs to pose a novel threat to poultry and wild birds (Figure 4).

Second, we found that H5N6 AIVs could be divided into Group 1 and Group 2 viruses (Figure 4). Group 2 H5N6 HPAIVs showed limited evolutionary activity because group 2 H5N6 virus endemics were “terminated” by the year 2014. In contrast, Group 1 H5N6 viruses continuously evolved, as shown by the following: (1) all of the most recently isolated H5N6 HPAIVs are clustered into this sub-lineage; (2) more frequent genetic reassortment (H5N1, H5N2, and H5N8) is observed in this group; (3) the HPAI H5N6 virus has a wider range of hosts, especially migratory birds and raptors, and this increases concern that H5N6 may spread to other regions of the world.

### Genetic characteristics of N6-subtype AIV isolates collected from 2012 to 2016

**Figure 5:**
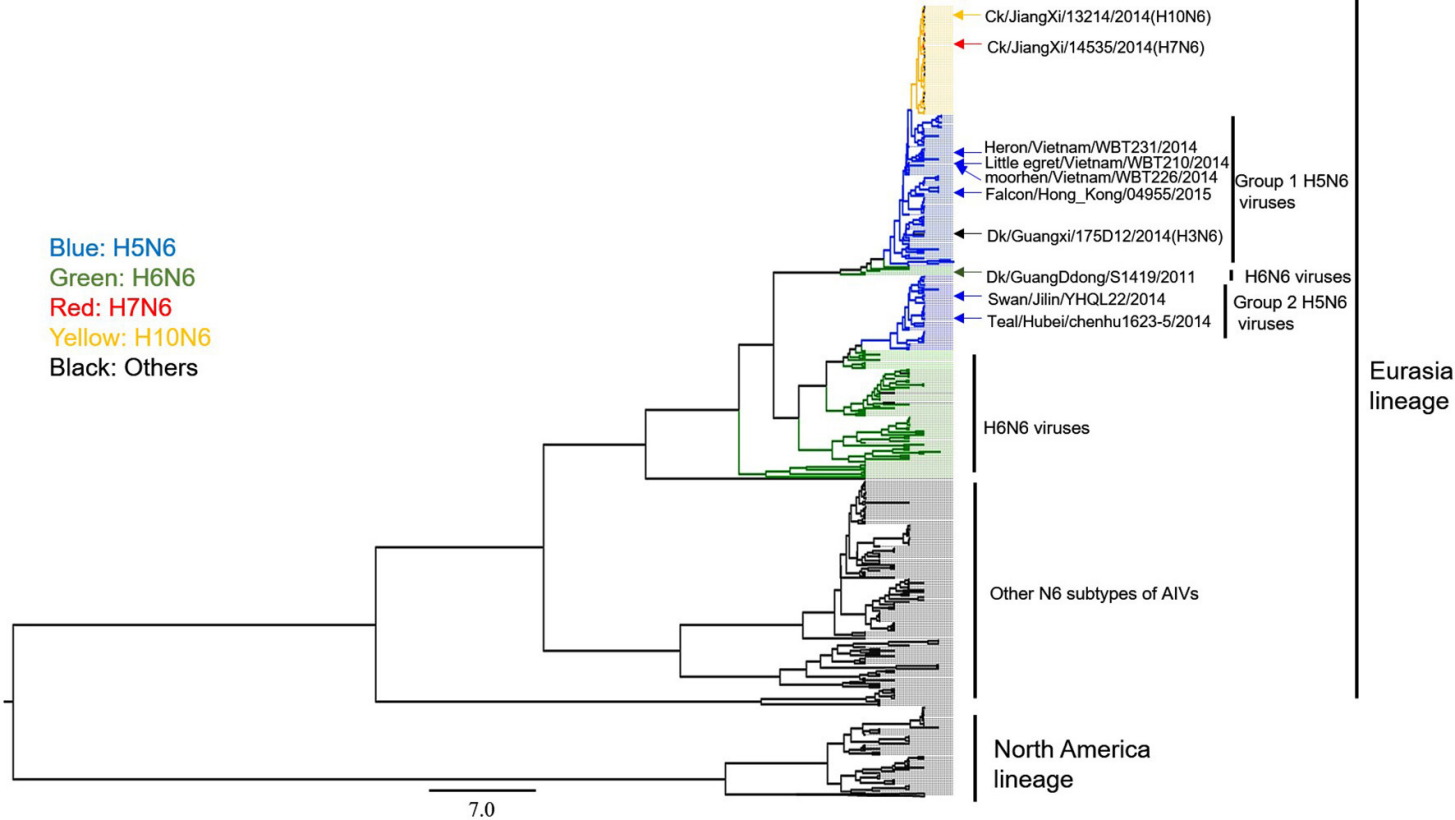
**Phylogenetic analysis of N6 subtype AIVs in Asia, Europe, and North America**. The MCC trees were built using BEAST (v1.8.4) and illustrated using FigTree (v1.4.2).

We next focused on the genetic analysis of N6 genes. We found that N6 subtype AIVs can be grouped into the following two lineages: Eurasian and North American (Figure 5). Most of the identified H5N6 isolates belonged to the Eurasian lineage. The H10N6 and H7N6 influenza viruses were closely related to the H5N6 viruses that are circulating in South China (Figure 5). These results indicate that H5N6 AIVs have undergone reassortment with the H10N8 and H7N9 viruses, consistent with previous studies (Lam et al., 2015; Ma et al., 2015). Additionally, there is no evidence showing that the N6 genes of H5N6 AIVs were transmitted to other continents. However, the Common Teal (*Anas crecca*), which has a wintering region that includes areas such as the Beringian Crucible (Alaska and the Russian Far East), has been identified as a host for the H5N6 influenza virus. The Beringian Crucible is an important channel of communication between the Eurasian and North American lineages (Lee et al., 2015). Evidence of reassortment among H5N8, H5N2, and H5N1 is rightfully increasing concern that H5N6 might become capable of spreading to other regions of the world.

## Discussion

HPAIV is a health challenge to poultry species, mammals, wild birds, and humans. These viruses cause considerable economic losses around the world. In our research, we reported an outbreak of HPAI H5N6 virus in a peafowl farm in Jiangxi Province, China. The isolated JA1/2016 virus was most probably transmitted to Jiangxi Province from Guangdong Province, and the estimated divergence time between JA1/2016 and the FG594-like virus was June 2014, indicating that JA1/2016 had undergone a long period of antigenic drift (Figure 3). Additionally, we found that HA and NA gene evolved faster than other gene segments, the results indicated that antigenic drift plays an important role in the adaption of H5N6 influenza viruses. The SkyGrid figure for the HA and NA genes suggested an increase in number of effective population size since the year 2013, which is in agreement with the emergence and dissemination of these triple-reassortant H5N6 AIVs (Supplementary Figure 3; Bi et al., 2015).

The continual evolution of H5N6 AIVs highlights the persistent threat that is posed by the influenza ecosystem of China. First, H5N6 HPAIVs have repeatedly caused outbreaks in the breeding industry (Supplementary Figure 1). Second, H5N6 HPAIVs have already spilled over into wild bird populations, which may result in the spread of these viruses to other continents during long-distance migrations (Figure 4). Third, the risk that H5N6 might cause a pandemic in humans is limited. Finally, the enzootic H5N1, H5N2, H5N8 viruses and H5N6 AIVs have been found to interact with the “viral gene pool” that is circulating in Asia, and this has given rise to novel variants, such as H10N6, H7N6, H3N6, and novel H5N8 (Figures 3, 4). Relatively little is known about H7N6 and H10N6 AIVs or their pathogenicity in mammals. It is difficult to assess their threat to humans. Moreover, many mixed isolates have been noted because of their homology to H10N6 viruses (Figure 4). These results suggest that chickens are extensively co-infected with AIVs that and long-term monitoring of AIVs in poultry is consequently necessary.

In fact, the low pathogenicity H5N6 influenza viruses has been noted in the past. For example, H5N6 was detected in Wisconsin in 1975, in Germany in 1984, and in California in 2013, but these outbreaks had little impact on the poultry industry or public health. In 2013, another H5N6 AIV (A/duck/Vietnam/LBM360c1-4-1/2013) was submitted to the NCBI database (GenBank: LC010696). Based on our analysis, this virus obtained its surface gene from A/duck/Khanhhoa/CWI-36/2014 (H5N1) and A/muscovy duck/Quang Ninh/71/2013(H6N6). The latter is closely related to the donor H6N6 virus, which is a Group 2 H5N6 influenza virus (Figure 4). Intriguingly, LBM360c1-4-1 and Group 2 AIVs then vanished for reasons that remain unclear.

We selected 122 strains of H5N6 viruses for our analysis. Among these, 66.39% of the viruses were derived from poultry (22 chickens, 47 ducks, and 12 geese), while 12 isolates were derived from eight species of migratory birds, and these were found to harbor H5N6 AIVs. These results indicated that the H5N6 virus has become entrenched in an ecological niche from which it may present a long-term pandemic threat to other regions of the world. Currently, H5N6 AIVs require additional adaptations to infect humans (Supplementary Table S3). However, their continual evolution and the extensive exposure of the human population to the H5N6 virus or its variants increases the risk that these viruses will acquire the ability to be transmitted from human-to-human.

Recent studies have revealed that the Beringian Crucible plays a role in influenza virus transmission (Ip et al., 2008; Pearce et al., 2011; Lee et al., 2015). Beringian Crucible is an important breeding ground for both New World and Old World migratory systems. It is estimated that approximately 1.5–2.9 million aquatic birds migrate from Asia to Alaska annually. These birds provide a potential channel by which AIVs could be transmitted intercontinentally. Evidence has demonstrated the successful transmission of the H5N8 virus from Eurasia to America (Lee et al., 2015). The Common Teal is a type of migratory bird that has been identified as a host of the H5N6 virus. The breeding region of the teal is widely distributed and spans the area from Siberia to the Far East and the Beringian Crucible. The risk that H5N6 could be transmitted to other continents is consequently increased. Overall, we suggest that scientists in other countries should pay more attention to the control and surveillance of the H5N6 influenza viruses.

A major limitation of this study is that our major conclusion regarding the origin of JA1/2016 and the genesis of Group 1 H5N6 AIVs is based solely on a phylogenetic analysis. Although phylogenetic analyses are widely used to trace the source of AIVs, overstatements regarding conclusions have been drawn by many researchers. Despite the controversy regarding these algorithms, the data that were used in most of our analyses were obtained over time from some, but not all, of the infected hosts (see figure 2.9 in the followed reference; Drummond and Bouckaert, 2015). For this reason, improved methods for determining sampling density (both space and time) are needed to more accurately evaluate AIV evolution.

## Author contributions

HH and ML designed this experiment, and wrote this manuscript; ML and NZ collected the samples and performed the experiments; JL, Yuan Li, LC, LZ, and JM participated in the data analysis; GY, CW, and YW provided some useful suggestion on the project and manuscript revising. Yanhua Liu collected this samples and was in charge of the animal experiment.

### Conflict of interest statement

The authors declare that the research was conducted in the absence of any commercial or financial relationships that could be construed as a potential conflict of interest.
