# Supplementary material for: Genetic Characterization of Continually Evolving Highly Pathogenic H5N6 Influenza Viruses in China, 2012–2016
Source: Front Microbiol. 2017 Feb 28;8:260. doi: 10.3389/fmicb.2017.00260 (PMC5329059; doi:10.3389/fmicb.2017.00260)
Supplement: Supplementary file 1 [file DataSheet1.pdf]

Supplementary Material

Genetic Characterization of Continually Evolving Highly Pathogenic H5N6 Influenza Viruses in China, 2012-2016

Meng Li[1,2], Na Zhao[1,2], Jing Luo[1], Yuan Li[3], Lin Chen[1,2], Jiajun Ma[1,2], Lin Zhao[1], Guohui Yuan[1] , Chengmin Wang[1], Yutian Wang[4], Yanhua Liu[4], Hongxuan He[1,†]

1 National Research Center for Wildlife Borne Diseases, Institute of Zoology, Chinese Academy of Sciences, Beijing, PR China

2 University of the Chinese Academy of Sciences, Beijing, PR China

3 Hebei Normal University of Science and Technology, Qinghuangdao, Hebei Province, PR China

4 Beijing Animal Husbandry Station, Beijing, PR China

† Corresponding author; Email: hehx@ioz.ac.cn

1 SUPPLEMENTARY FIGURES AND TABLES

1.1 SUPPLEMENTARY FIGURES

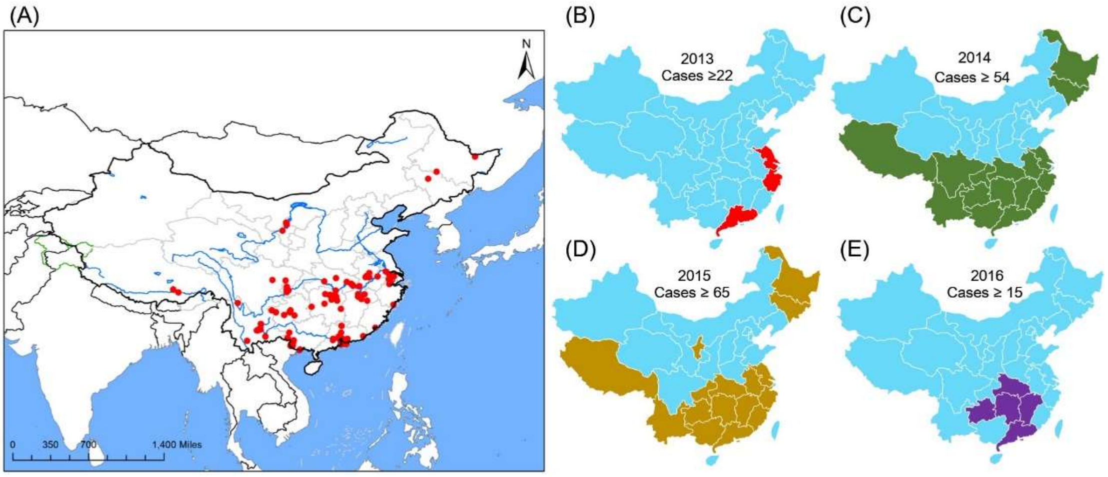

Supplementary Figure 1. Statistical analysis of H5N6 outbreaks in China. (A) H5N6 AIVs repeatedly outbroke in China. (B) to (E) The number and distribution of H5N6 outbreaks in each year. The data were obtained from FAO EMPRES-i (http://empres-i.fao.org).

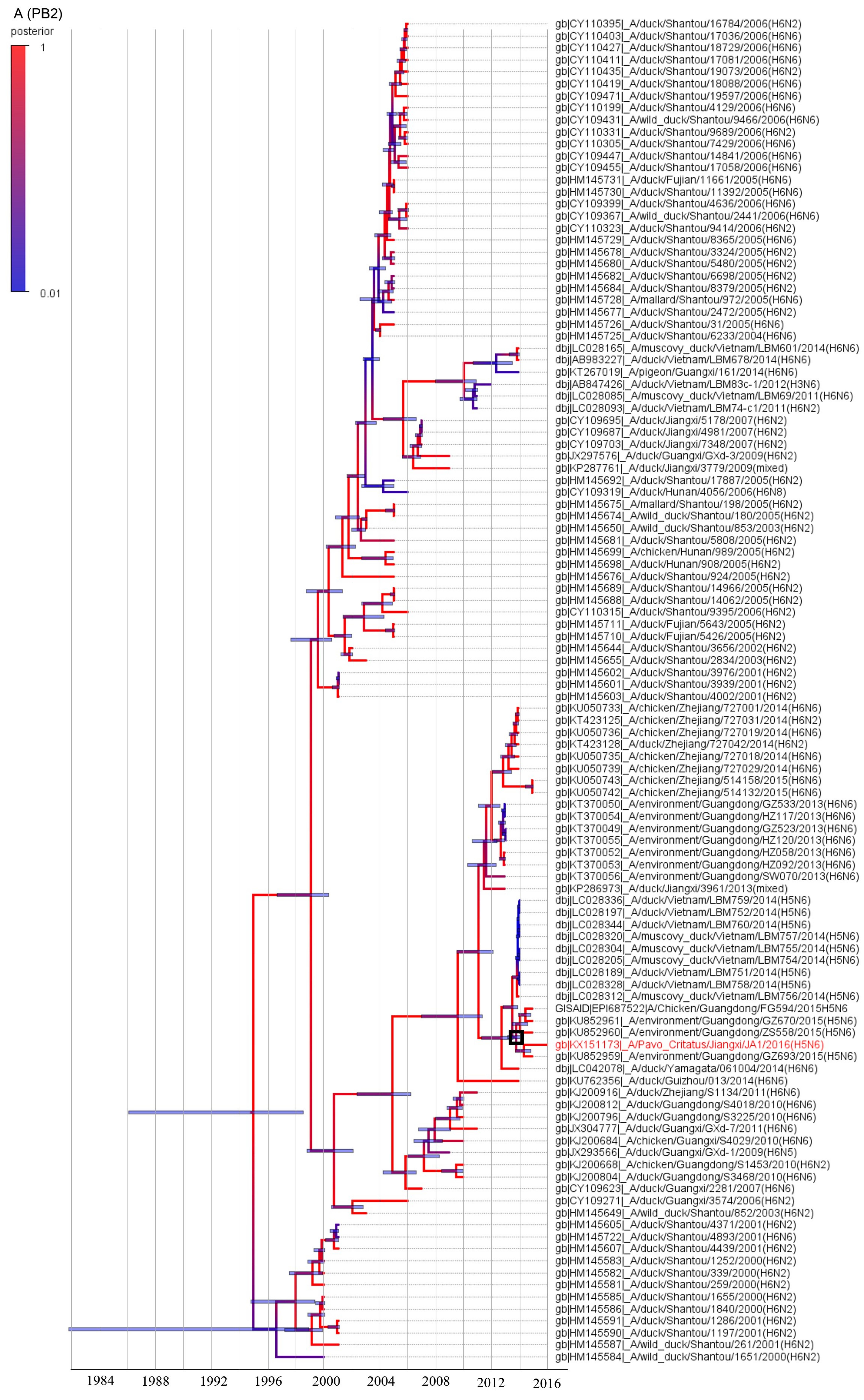

A (PB2)
posterior
1
0.01
gb|CY110395|_A/duck/Shantou/16784/2006(H6N2)
gb|CY110403|_A/duck/Shantou/17036/2006(H6N6)
gb|CY110427|_A/duck/Shantou/18729/2006(H6N6)
gb|CY110411|_A/duck/Shantou/17081/2006(H6N6)
gb|CY110435|_A/duck/Shantou/19073/2006(H6N2)
gb|CY110419|_A/duck/Shantou/18088/2006(H6N6)
gb|CY109471|_A/duck/Shantou/19597/2006(H6N6)
gb|CY110199|_A/duck/Shantou/4129/2006(H6N6)
gb|CY109431|_A/wild_duck/Shantou/9466/2006(H6N6)
gb|CY110331|_A/duck/Shantou/9689/2006(H6N2)
gb|CY110305|_A/duck/Shantou/7429/2006(H6N6)
gb|CY109447|_A/duck/Shantou/14841/2006(H6N6)
gb|CY109455|_A/duck/Shantou/17058/2006(H6N6)
gb|HM145731|_A/duck/Fujian/11661/2005(H6N6)
gb|HM145730|_A/duck/Shantou/11392/2005(H6N6)
gb|CY109399|_A/duck/Shantou/4636/2006(H6N6)
gb|CY109367|_A/wild_duck/Shantou/2441/2006(H6N6)
gb|CY110323|_A/duck/Shantou/9414/2006(H6N2)
gb|HM145729|_A/duck/Shantou/8365/2005(H6N6)
gb|HM145678|_A/duck/Shantou/3324/2005(H6N2)
gb|HM145680|_A/duck/Shantou/5480/2005(H6N2)
gb|HM145682|_A/duck/Shantou/6698/2005(H6N2)
gb|HM145684|_A/duck/Shantou/8379/2005(H6N2)
gb|HM145728|_A/mallard/Shantou/972/2005(H6N6)
gb|HM145677|_A/duck/Shantou/2472/2005(H6N2)
gb|HM145726|_A/duck/Shantou/31/2005(H6N6)
gb|HM145725|_A/duck/Shantou/6233/2004(H6N6)
dbj|LC028165|_A/muscovy_duck/Vietnam/LBM601/2014(H6N6)
dbj|AB983227|_A/duck/Vietnam/LBM678/2014(H6N6)
gb|KT267019|_A/pigeon/Guangxi/161/2014(H6N6)
dbj|AB847426|_A/duck/Vietnam/LBM83c-1/2012(H3N6)
dbj|LC028085|_A/muscovy_duck/Vietnam/LBM69/2011(H6N6)
dbj|LC028093|_A/duck/Vietnam/LBM74-c1/2011(H6N2)
gb|CY109695|_A/duck/Jiangxi/5178/2007(H6N2)
gb|CY109687|_A/duck/Jiangxi/4981/2007(H6N2)
gb|CY109703|_A/duck/Jiangxi/7348/2007(H6N2)
gb|JX297576|_A/duck/Guangxi/GXd-3/2009(H6N2)
gb|KP287761|_A/duck/Jiangxi/3779/2009(mixed)
gb|HM145692|_A/duck/Shantou/17887/2005(H6N2)
gb|CY109319|_A/duck/Hunan/4056/2006(H6N8)
gb|HM145675|_A/mallard/Shantou/198/2005(H6N2)
gb|HM145674|_A/wild_duck/Shantou/180/2005(H6N2)
gb|HM145650|_A/wild_duck/Shantou/853/2003(H6N2)
gb|HM145681|_A/duck/Shantou/5808/2005(H6N2)
gb|HM145699|_A/chicken/Hunan/989/2005(H6N2)
gb|HM145698|_A/duck/Hunan/908/2005(H6N2)
gb|HM145676|_A/duck/Shantou/924/2005(H6N2)
gb|HM145689|_A/duck/Shantou/14966/2005(H6N2)
gb|HM145688|_A/duck/Shantou/14062/2005(H6N2)
gb|CY110315|_A/duck/Shantou/9395/2006(H6N2)
gb|HM145711|_A/duck/Fujian/5643/2005(H6N2)
gb|HM145710|_A/duck/Fujian/5426/2005(H6N2)
gb|HM145644|_A/duck/Shantou/3656/2002(H6N2)
gb|HM145655|_A/duck/Shantou/2834/2003(H6N2)
gb|HM145602|_A/duck/Shantou/3976/2001(H6N2)
gb|HM145601|_A/duck/Shantou/3939/2001(H6N2)
gb|HM145603|_A/duck/Shantou/4002/2001(H6N2)
gb|KU050733|_A/chicken/Zhejiang/727001/2014(H6N6)
gb|KT423125|_A/chicken/Zhejiang/727031/2014(H6N2)
gb|KU050736|_A/chicken/Zhejiang/727019/2014(H6N6)
gb|KT423128|_A/duck/Zhejiang/727042/2014(H6N2)
gb|KU050735|_A/chicken/Zhejiang/727018/2014(H6N6)
gb|KU050739|_A/chicken/Zhejiang/727029/2014(H6N6)
gb|KU050743|_A/chicken/Zhejiang/514158/2015(H6N6)
gb|KU050742|_A/chicken/Zhejiang/514132/2015(H6N6)
gb|KT370050|_A/environment/Guangdong/GZ533/2013(H6N6)
gb|KT370054|_A/environment/Guangdong/HZ117/2013(H6N6)
gb|KT370049|_A/environment/Guangdong/GZ523/2013(H6N6)
gb|KT370055|_A/environment/Guangdong/HZ120/2013(H6N6)
gb|KT370052|_A/environment/Guangdong/HZ058/2013(H6N6)
gb|KT370053|_A/environment/Guangdong/HZ092/2013(H6N6)
gb|KT370056|_A/environment/Guangdong/SW070/2013(H6N6)
gb|KP286973|_A/duck/Jiangxi/3961/2013(mixed)
dbj|LC028336|_A/duck/Vietnam/LBM759/2014(H5N6)
dbj|LC028197|_A/duck/Vietnam/LBM752/2014(H5N6)
dbj|LC028344|_A/duck/Vietnam/LBM760/2014(H5N6)
dbj|LC028320|_A/muscovy_duck/Vietnam/LBM757/2014(H5N6)
dbj|LC028304|_A/muscovy_duck/Vietnam/LBM755/2014(H5N6)
dbj|LC028205|_A/muscovy_duck/Vietnam/LBM754/2014(H5N6)
dbj|LC028189|_A/duck/Vietnam/LBM751/2014(H5N6)
dbj|LC028328|_A/duck/Vietnam/LBM758/2014(H5N6)
dbj|LC028312|_A/muscovy_duck/Vietnam/LBM756/2014(H5N6)
GISAID|EPI687522|A/Chicken/Guangdong/FG594/2015H5N6
gb|KU852961|_A/environment/Guangdong/GZ670/2015(H5N6)
gb|KU852960|_A/environment/Guangdong/ZS558/2015(H5N6)
gb|KX151173|_A/Pavo_Critatus/Jiangxi/JA1/2016(H5N6)
gb|KU852959|_A/environment/Guangdong/GZ693/2015(H5N6)
dbj|LC042078|_A/duck/Yamagata/061004/2014(H6N6)
gb|KU762356|_A/duck/Guizhou/013/2014(H6N6)
gb|KJ200916|_A/duck/Zhejiang/S1134/2011(H6N6)
gb|KJ200812|_A/duck/Guangdong/S4018/2010(H6N6)
gb|KJ200796|_A/duck/Guangdong/S3225/2010(H6N6)
gb|JX304777|_A/duck/Guangxi/GXd-7/2011(H6N6)
gb|KJ200684|_A/chicken/Guangxi/S4029/2010(H6N6)
gb|JX293566|_A/duck/Guangxi/GXd-1/2009(H6N5)
gb|KJ200668|_A/chicken/Guangdong/S1453/2010(H6N2)
gb|KJ200804|_A/duck/Guangdong/S3468/2010(H6N6)
gb|CY109623|_A/duck/Guangxi/2281/2007(H6N6)
gb|CY109271|_A/duck/Guangxi/3574/2006(H6N2)
gb|HM145649|_A/wild_duck/Shantou/852/2003(H6N2)
gb|HM145605|_A/duck/Shantou/4371/2001(H6N2)
gb|HM145722|_A/duck/Shantou/4893/2001(H6N6)
gb|HM145607|_A/duck/Shantou/4439/2001(H6N2)
gb|HM145583|_A/duck/Shantou/1252/2000(H6N2)
gb|HM145582|_A/duck/Shantou/339/2000(H6N2)
gb|HM145581|_A/duck/Shantou/259/2000(H6N2)
gb|HM145585|_A/duck/Shantou/1655/2000(H6N2)
gb|HM145586|_A/duck/Shantou/1840/2000(H6N2)
gb|HM145591|_A/duck/Shantou/1286/2001(H6N2)
gb|HM145590|_A/duck/Shantou/1197/2001(H6N2)
gb|HM145587|_A/wild_duck/Shantou/261/2001(H6N2)
gb|HM145584|_A/wild_duck/Shantou/1651/2000(H6N2)
1984
1988
1992
1996
2000
2004
2008
2012
2016

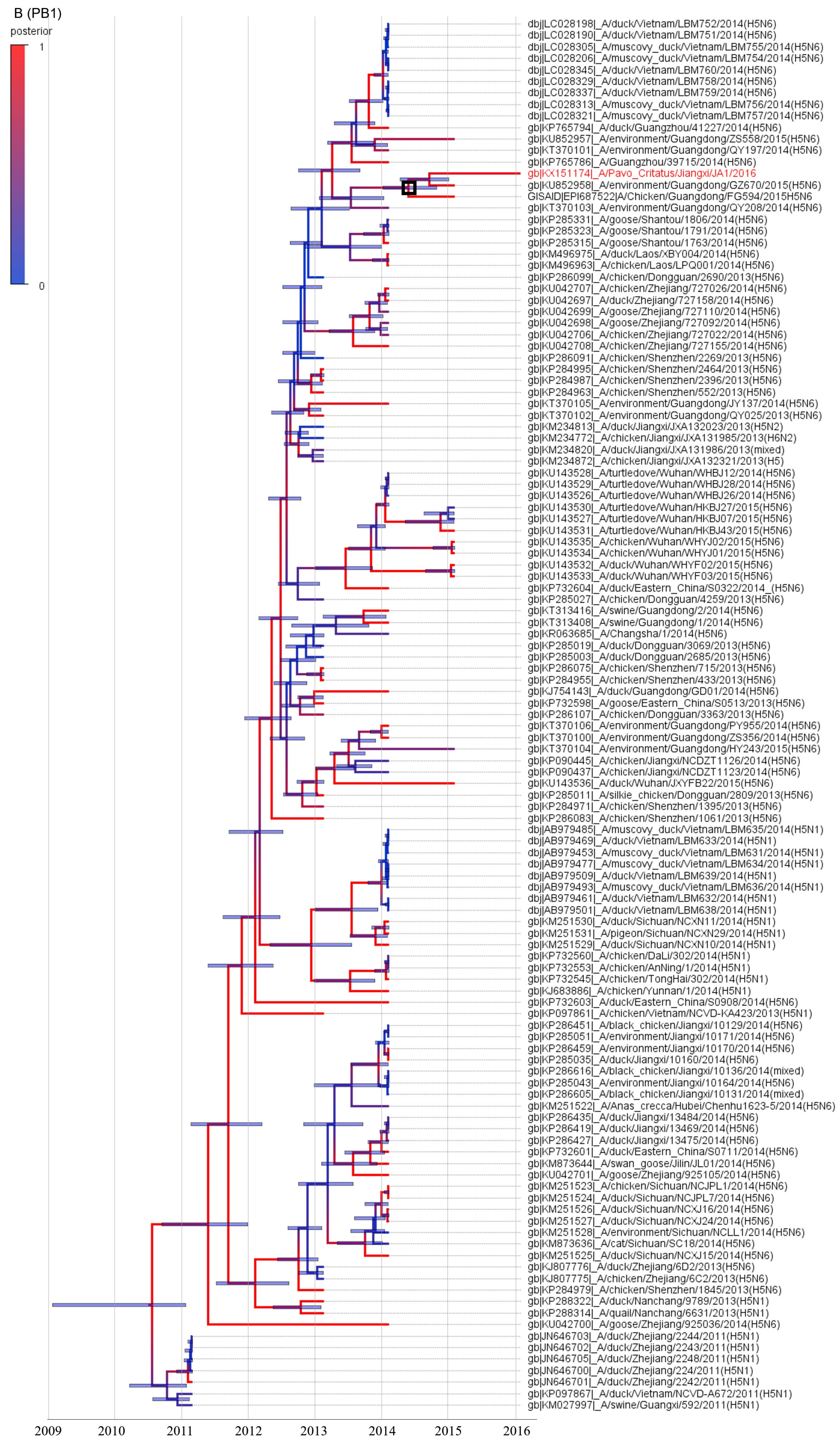

B (PB1)
posterior
1
0
dbj|LC028198|_A/duck/Vietnam/LBM752/2014(H5N6)
dbj|LC028190|_A/duck/Vietnam/LBM751/2014(H5N6)
dbj|LC028305|_A/muscovy_duck/Vietnam/LBM755/2014(H5N6)
dbj|LC028206|_A/muscovy_duck/Vietnam/LBM754/2014(H5N6)
dbj|LC028345|_A/duck/Vietnam/LBM760/2014(H5N6)
dbj|LC028329|_A/duck/Vietnam/LBM758/2014(H5N6)
dbj|LC028337|_A/duck/Vietnam/LBM759/2014(H5N6)
dbj|LC028313|_A/muscovy_duck/Vietnam/LBM756/2014(H5N6)
dbj|LC028321|_A/muscovy_duck/Vietnam/LBM757/2014(H5N6)
gb|KP765794|_A/duck/Guangzhou/41227/2014(H5N6)
gb|KU852957|_A/environment/Guangdong/ZS558/2015(H5N6)
gb|KT370101|_A/environment/Guangdong/QY197/2014(H5N6)
gb|KP765786|_A/Guangzhou/39715/2014(H5N6)
gb|KX151174|_A/Pavo_Critatus/Jiangxi/JA1/2016
gb|KU852958|_A/environment/Guangdong/GZ670/2015(H5N6)
GISAID|EPI687522|A/Chicken/Guangdong/FG594/2015H5N6
gb|KT370103|_A/environment/Guangdong/QY208/2014(H5N6)
gb|KP285331|_A/goose/Shantou/1806/2014(H5N6)
gb|KP285323|_A/goose/Shantou/1791/2014(H5N6)
gb|KP285315|_A/goose/Shantou/1763/2014(H5N6)
gb|KM496975|_A/duck/Laos/XBY004/2014(H5N6)
gb|KM496963|_A/chicken/Laos/LPQ001/2014(H5N6)
gb|KP286099|_A/chicken/Dongguan/2690/2013(H5N6)
gb|KU042707|_A/chicken/Zhejiang/727026/2014(H5N6)
gb|KU042697|_A/duck/Zhejiang/727158/2014(H5N6)
gb|KU042699|_A/goose/Zhejiang/727110/2014(H5N6)
gb|KU042698|_A/goose/Zhejiang/727092/2014(H5N6)
gb|KU042706|_A/chicken/Zhejiang/727022/2014(H5N6)
gb|KU042708|_A/chicken/Zhejiang/727155/2014(H5N6)
gb|KP286091|_A/chicken/Shenzhen/2269/2013(H5N6)
gb|KP284995|_A/chicken/Shenzhen/2464/2013(H5N6)
gb|KP284987|_A/chicken/Shenzhen/2396/2013(H5N6)
gb|KP284963|_A/chicken/Shenzhen/552/2013(H5N6)
gb|KT370105|_A/environment/Guangdong/JY137/2014(H5N6)
gb|KT370102|_A/environment/Guangdong/QY025/2013(H5N6)
gb|KM234813|_A/duck/Jiangxi/JXA132023/2013(H5N2)
gb|KM234772|_A/chicken/Jiangxi/JXA131985/2013(H6N2)
gb|KM234820|_A/duck/Jiangxi/JXA131986/2013(mixed)
gb|KM234872|_A/chicken/Jiangxi/JXA132321/2013(H5)
gb|KU143528|_A/turtledove/Wuhan/WHBJ12/2014(H5N6)
gb|KU143529|_A/turtledove/Wuhan/WHBJ28/2014(H5N6)
gb|KU143526|_A/turtledove/Wuhan/WHBJ26/2014(H5N6)
gb|KU143530|_A/turtledove/Wuhan/HKBJ27/2015(H5N6)
gb|KU143527|_A/turtledove/Wuhan/HKBJ07/2015(H5N6)
gb|KU143531|_A/turtledove/Wuhan/HKBJ43/2015(H5N6)
gb|KU143535|_A/chicken/Wuhan/WHYJ02/2015(H5N6)
gb|KU143534|_A/chicken/Wuhan/WHYJ01/2015(H5N6)
gb|KU143532|_A/duck/Wuhan/WHYF02/2015(H5N6)
gb|KU143533|_A/duck/Wuhan/WHYF03/2015(H5N6)
gb|KP732604|_A/duck/Eastern_China/S0322/2014_(H5N6)
gb|KP285027|_A/chicken/Dongguan/4259/2013(H5N6)
gb|KT313416|_A/swine/Guangdong/2/2014(H5N6)
gb|KT313408|_A/swine/Guangdong/1/2014(H5N6)
gb|KR063685|_A/Changsha/1/2014(H5N6)
gb|KP285019|_A/duck/Dongguan/3069/2013(H5N6)
gb|KP285003|_A/duck/Dongguan/2685/2013(H5N6)
gb|KP286075|_A/chicken/Shenzhen/715/2013(H5N6)
gb|KP284955|_A/chicken/Shenzhen/433/2013(H5N6)
gb|KJ754143|_A/duck/Guangdong/GD01/2014(H5N6)
gb|KP732598|_A/goose/Eastern_China/S0513/2013(H5N6)
gb|KP286107|_A/chicken/Dongguan/3363/2013(H5N6)
gb|KT370106|_A/environment/Guangdong/PY955/2014(H5N6)
gb|KT370100|_A/environment/Guangdong/ZS356/2014(H5N6)
gb|KT370104|_A/environment/Guangdong/HY243/2015(H5N6)
gb|KP090445|_A/chicken/Jiangxi/NCDZT1126/2014(H5N6)
gb|KP090437|_A/chicken/Jiangxi/NCDZT1123/2014(H5N6)
gb|KU143536|_A/duck/Wuhan/JXYFB22/2015(H5N6)
gb|KP285011|_A/silkie_chicken/Dongguan/2809/2013(H5N6)
gb|KP284971|_A/chicken/Shenzhen/1395/2013(H5N6)
gb|KP286083|_A/chicken/Shenzhen/1061/2013(H5N6)
dbj|AB979485|_A/muscovy_duck/Vietnam/LBM635/2014(H5N1)
dbj|AB979469|_A/duck/Vietnam/LBM633/2014(H5N1)
dbj|AB979453|_A/muscovy_duck/Vietnam/LBM631/2014(H5N1)
dbj|AB979477|_A/muscovy_duck/Vietnam/LBM634/2014(H5N1)
dbj|AB979509|_A/duck/Vietnam/LBM639/2014(H5N1)
dbj|AB979493|_A/muscovy_duck/Vietnam/LBM636/2014(H5N1)
dbj|AB979461|_A/duck/Vietnam/LBM632/2014(H5N1)
dbj|AB979501|_A/duck/Vietnam/LBM638/2014(H5N1)
gb|KM251530|_A/duck/Sichuan/NCXN11/2014(H5N1)
gb|KM251531|_A/pigeon/Sichuan/NCXN29/2014(H5N1)
gb|KM251529|_A/duck/Sichuan/NCXN10/2014(H5N1)
gb|KP732560|_A/chicken/DaLi/302/2014(H5N1)
gb|KP732553|_A/chicken/AnNing/1/2014(H5N1)
gb|KP732545|_A/chicken/TongHai/302/2014(H5N1)
gb|KJ683886|_A/chicken/Yunnan/1/2014(H5N1)
gb|KP732603|_A/duck/Eastern_China/S0908/2014(H5N6)
gb|KP097861|_A/chicken/Vietnam/NCVD-KA423/2013(H5N1)
gb|KP286451|_A/black_chicken/Jiangxi/10129/2014(H5N6)
gb|KP285051|_A/environment/Jiangxi/10171/2014(H5N6)
gb|KP286459|_A/environment/Jiangxi/10170/2014(H5N6)
gb|KP285035|_A/duck/Jiangxi/10160/2014(H5N6)
gb|KP286616|_A/black_chicken/Jiangxi/10136/2014(mixed)
gb|KP285043|_A/environment/Jiangxi/10164/2014(H5N6)
gb|KP286605|_A/black_chicken/Jiangxi/10131/2014(mixed)
gb|KM251522|_A/Anas_crecca/Hubei/Chenhu1623-5/2014(H5N6)
gb|KP286435|_A/duck/Jiangxi/13484/2014(H5N6)
gb|KP286419|_A/duck/Jiangxi/13469/2014(H5N6)
gb|KP286427|_A/duck/Jiangxi/13475/2014(H5N6)
gb|KP732601|_A/duck/Eastern_China/S0711/2014(H5N6)
gb|KM873644|_A/swan_goose/Jilin/JL01/2014(H5N6)
gb|KU042701|_A/goose/Zhejiang/925105/2014(H5N6)
gb|KM251523|_A/chicken/Sichuan/NCJPL1/2014(H5N6)
gb|KM251524|_A/duck/Sichuan/NCJPL7/2014(H5N6)
gb|KM251526|_A/duck/Sichuan/NCXJ16/2014(H5N6)
gb|KM251527|_A/duck/Sichuan/NCXJ24/2014(H5N6)
gb|KM251528|_A/environment/Sichuan/NCLL1/2014(H5N6)
gb|KM873636|_A/cat/Sichuan/SC18/2014(H5N6)
gb|KM251525|_A/duck/Sichuan/NCXJ15/2014(H5N6)
gb|KJ807776|_A/duck/Zhejiang/6D2/2013(H5N6)
gb|KJ807775|_A/chicken/Zhejiang/6C2/2013(H5N6)
gb|KP284979|_A/chicken/Shenzhen/1845/2013(H5N6)
gb|KP288322|_A/duck/Nanchang/9789/2013(H5N1)
gb|KP288314|_A/quail/Nanchang/6631/2013(H5N1)
gb|KU042700|_A/goose/Zhejiang/925036/2014(H5N6)
gb|JN646703|_A/duck/Zhejiang/2244/2011(H5N1)
gb|JN646702|_A/duck/Zhejiang/2243/2011(H5N1)
gb|JN646705|_A/duck/Zhejiang/2248/2011(H5N1)
gb|JN646700|_A/duck/Zhejiang/224/2011(H5N1)
gb|JN646701|_A/duck/Zhejiang/2242/2011(H5N1)
gb|KP097867|_A/duck/Vietnam/NCVD-A672/2011(H5N1)
gb|KM027997|_A/swine/Guangxi/592/2011(H5N1)
2009
2010
2011
2012
2013
2014
2015
2016

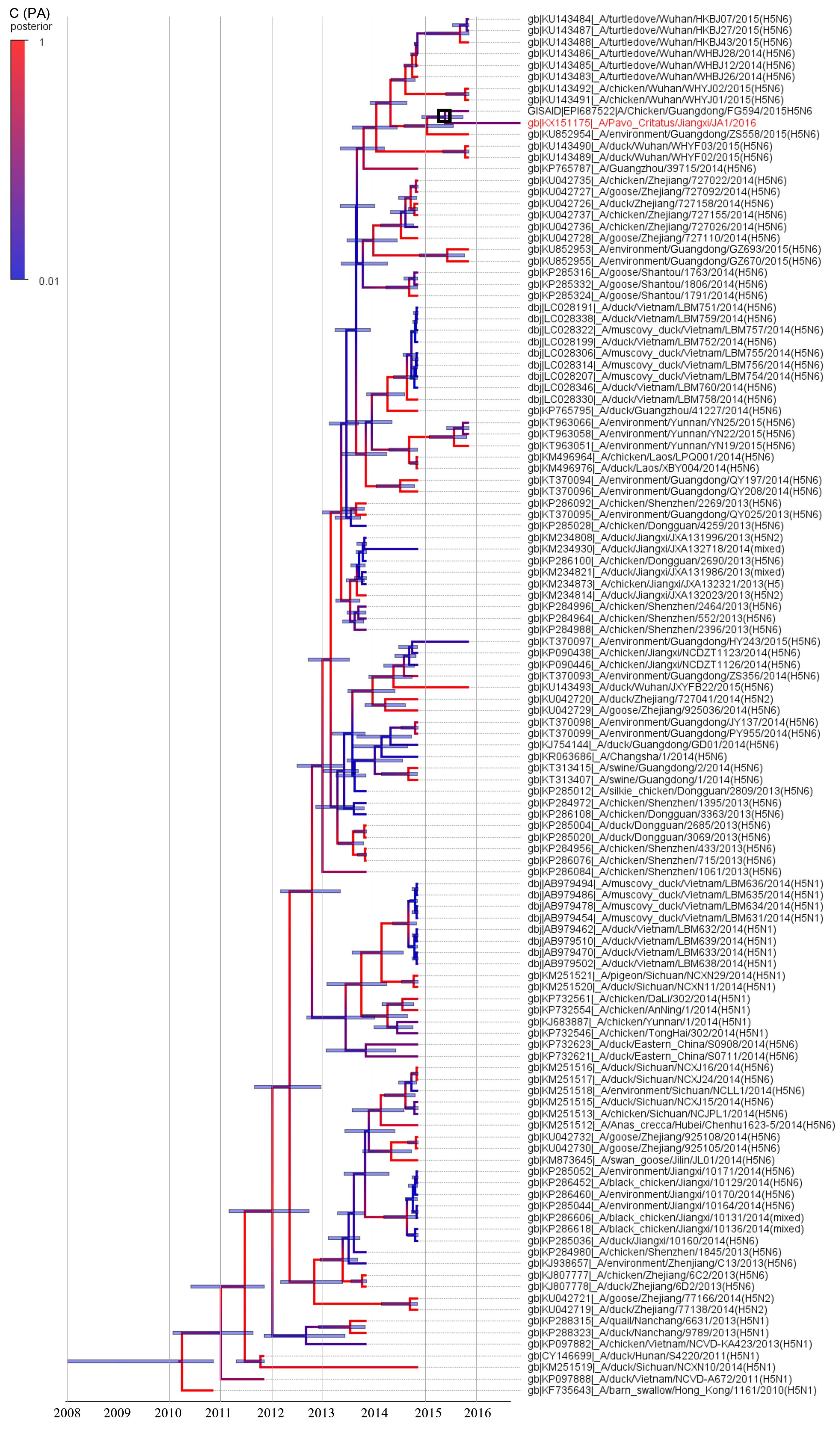
C (PA)
posterior
1
0.01
gb|KU143484|_A/turtledove/Wuhan/HKBJ07/2015(H5N6)
gb|KU143487|_A/turtledove/Wuhan/HKBJ27/2015(H5N6)
gb|KU143488|_A/turtledove/Wuhan/HKBJ43/2015(H5N6)
gb|KU143486|_A/turtledove/Wuhan/WHBJ28/2014(H5N6)
gb|KU143485|_A/turtledove/Wuhan/WHBJ12/2014(H5N6)
gb|KU143483|_A/turtledove/Wuhan/WHBJ26/2014(H5N6)
gb|KU143492|_A/chicken/Wuhan/WHYJ02/2015(H5N6)
gb|KU143491|_A/chicken/Wuhan/WHYJ01/2015(H5N6)
GISAID|EPI687522|A/Chicken/Guangdong/FG594/2015H5N6
gb|KX151175|_A/Pavo_Critatus/Jiangxi/JA1/2016
gb|KU852954|_A/environment/Guangdong/ZS558/2015(H5N6)
gb|KU143490|_A/duck/Wuhan/WHYF03/2015(H5N6)
gb|KU143489|_A/duck/Wuhan/WHYF02/2015(H5N6)
gb|KP765787|_A/Guangzhou/39715/2014(H5N6)
gb|KU042735|_A/chicken/Zhejiang/727022/2014(H5N6)
gb|KU042727|_A/goose/Zhejiang/727092/2014(H5N6)
gb|KU042726|_A/duck/Zhejiang/727158/2014(H5N6)
gb|KU042737|_A/chicken/Zhejiang/727155/2014(H5N6)
gb|KU042736|_A/chicken/Zhejiang/727026/2014(H5N6)
gb|KU042728|_A/goose/Zhejiang/727110/2014(H5N6)
gb|KU852953|_A/environment/Guangdong/GZ693/2015(H5N6)
gb|KU852955|_A/environment/Guangdong/GZ670/2015(H5N6)
gb|KP285316|_A/goose/Shantou/1763/2014(H5N6)
gb|KP285332|_A/goose/Shantou/1806/2014(H5N6)
gb|KP285324|_A/goose/Shantou/1791/2014(H5N6)
dbj|LC028191|_A/duck/Vietnam/LBM751/2014(H5N6)
dbj|LC028338|_A/duck/Vietnam/LBM759/2014(H5N6)
dbj|LC028322|_A/muscovy_duck/Vietnam/LBM757/2014(H5N6)
dbj|LC028199|_A/duck/Vietnam/LBM752/2014(H5N6)
dbj|LC028306|_A/muscovy_duck/Vietnam/LBM755/2014(H5N6)
dbj|LC028314|_A/muscovy_duck/Vietnam/LBM756/2014(H5N6)
dbj|LC028207|_A/muscovy_duck/Vietnam/LBM754/2014(H5N6)
dbj|LC028346|_A/duck/Vietnam/LBM760/2014(H5N6)
dbj|LC028330|_A/duck/Vietnam/LBM758/2014(H5N6)
gb|KP765795|_A/duck/Guangzhou/41227/2014(H5N6)
gb|KT963066|_A/environment/Yunnan/YN25/2015(H5N6)
gb|KT963058|_A/environment/Yunnan/YN22/2015(H5N6)
gb|KT963051|_A/environment/Yunnan/YN19/2015(H5N6)
gb|KM496964|_A/chicken/Laos/LPQ001/2014(H5N6)
gb|KM496976|_A/duck/Laos/XBY004/2014(H5N6)
gb|KT370094|_A/environment/Guangdong/QY197/2014(H5N6)
gb|KT370096|_A/environment/Guangdong/QY208/2014(H5N6)
gb|KP286092|_A/chicken/Shenzhen/2269/2013(H5N6)
gb|KT370095|_A/environment/Guangdong/QY025/2013(H5N6)
gb|KP285028|_A/chicken/Dongguan/4259/2013(H5N6)
gb|KM234808|_A/duck/Jiangxi/JXA131996/2013(H5N2)
gb|KM234930|_A/duck/Jiangxi/JXA132718/2014(mixed)
gb|KP286100|_A/chicken/Dongguan/2690/2013(H5N6)
gb|KM234821|_A/duck/Jiangxi/JXA131986/2013(mixed)
gb|KM234873|_A/chicken/Jiangxi/JXA132321/2013(H5)
gb|KM234814|_A/duck/Jiangxi/JXA132023/2013(H5N2)
gb|KP284996|_A/chicken/Shenzhen/2464/2013(H5N6)
gb|KP284964|_A/chicken/Shenzhen/552/2013(H5N6)
gb|KP284988|_A/chicken/Shenzhen/2396/2013(H5N6)
gb|KT370097|_A/environment/Guangdong/HY243/2015(H5N6)
gb|KP090438|_A/chicken/Jiangxi/NCDZT1123/2014(H5N6)
gb|KP090446|_A/chicken/Jiangxi/NCDZT1126/2014(H5N6)
gb|KT370093|_A/environment/Guangdong/ZS356/2014(H5N6)
gb|KU143493|_A/duck/Wuhan/JXYFB22/2015(H5N6)
gb|KU042720|_A/duck/Zhejiang/727041/2014(H5N2)
gb|KU042729|_A/goose/Zhejiang/925036/2014(H5N6)
gb|KT370098|_A/environment/Guangdong/JY137/2014(H5N6)
gb|KT370099|_A/environment/Guangdong/PY955/2014(H5N6)
gb|KJ754144|_A/duck/Guangdong/GD01/2014(H5N6)
gb|KR063686|_A/Changsha/1/2014(H5N6)
gb|KT313415|_A/swine/Guangdong/2/2014(H5N6)
gb|KT313407|_A/swine/Guangdong/1/2014(H5N6)
gb|KP285012|_A/silkie_chicken/Dongguan/2809/2013(H5N6)
gb|KP284972|_A/chicken/Shenzhen/1395/2013(H5N6)
gb|KP286108|_A/chicken/Dongguan/3363/2013(H5N6)
gb|KP285004|_A/duck/Dongguan/2685/2013(H5N6)
gb|KP285020|_A/duck/Dongguan/3069/2013(H5N6)
gb|KP284956|_A/chicken/Shenzhen/433/2013(H5N6)
gb|KP286076|_A/chicken/Shenzhen/715/2013(H5N6)
gb|KP286084|_A/chicken/Shenzhen/1061/2013(H5N6)
dbj|AB979494|_A/muscovy_duck/Vietnam/LBM636/2014(H5N1)
dbj|AB979486|_A/muscovy_duck/Vietnam/LBM635/2014(H5N1)
dbj|AB979478|_A/muscovy_duck/Vietnam/LBM634/2014(H5N1)
dbj|AB979454|_A/muscovy_duck/Vietnam/LBM631/2014(H5N1)
dbj|AB979462|_A/duck/Vietnam/LBM632/2014(H5N1)
dbj|AB979510|_A/duck/Vietnam/LBM639/2014(H5N1)
dbj|AB979470|_A/duck/Vietnam/LBM633/2014(H5N1)
dbj|AB979502|_A/duck/Vietnam/LBM638/2014(H5N1)
gb|KM251521|_A/pigeon/Sichuan/NCXN29/2014(H5N1)
gb|KM251520|_A/duck/Sichuan/NCXN11/2014(H5N1)
gb|KP732561|_A/chicken/DaLi/302/2014(H5N1)
gb|KP732554|_A/chicken/AnNing/1/2014(H5N1)
gb|KJ683887|_A/chicken/Yunnan/1/2014(H5N1)
gb|KP732546|_A/chicken/TongHai/302/2014(H5N1)
gb|KP732623|_A/duck/Eastern_China/S0908/2014(H5N6)
gb|KP732621|_A/duck/Eastern_China/S0711/2014(H5N6)
gb|KM251516|_A/duck/Sichuan/NCXJ16/2014(H5N6)
gb|KM251517|_A/duck/Sichuan/NCXJ24/2014(H5N6)
gb|KM251518|_A/environment/Sichuan/NCLL1/2014(H5N6)
gb|KM251515|_A/duck/Sichuan/NCXJ15/2014(H5N6)
gb|KM251513|_A/chicken/Sichuan/NCJPL1/2014(H5N6)
gb|KM251512|_A/Anas_crecca/Hubei/Chenhu1623-5/2014(H5N6)
gb|KU042732|_A/goose/Zhejiang/925108/2014(H5N6)
gb|KU042730|_A/goose/Zhejiang/925105/2014(H5N6)
gb|KM873645|_A/swan_goose/Jilin/JL01/2014(H5N6)
gb|KP285052|_A/environment/Jiangxi/10171/2014(H5N6)
gb|KP286452|_A/black_chicken/Jiangxi/10129/2014(H5N6)
gb|KP286460|_A/environment/Jiangxi/10170/2014(H5N6)
gb|KP285044|_A/environment/Jiangxi/10164/2014(H5N6)
gb|KP286606|_A/black_chicken/Jiangxi/10131/2014(mixed)
gb|KP286618|_A/black_chicken/Jiangxi/10136/2014(mixed)
gb|KP285036|_A/duck/Jiangxi/10160/2014(H5N6)
gb|KP284980|_A/chicken/Shenzhen/1845/2013(H5N6)
gb|KJ938657|_A/environment/Zhenjiang/C13/2013(H5N6)
gb|KJ807777|_A/chicken/Zhejiang/6C2/2013(H5N6)
gb|KJ807778|_A/duck/Zhejiang/6D2/2013(H5N6)
gb|KU042721|_A/goose/Zhejiang/77166/2014(H5N2)
gb|KU042719|_A/duck/Zhejiang/77138/2014(H5N2)
gb|KP288315|_A/quail/Nanchang/6631/2013(H5N1)
gb|KP288323|_A/duck/Nanchang/9789/2013(H5N1)
gb|KP097882|_A/chicken/Vietnam/NCVD-KA423/2013(H5N1)
gb|CY146699|_A/duck/Hunan/S4220/2011(H5N1)
gb|KM251519|_A/duck/Sichuan/NCXN10/2014(H5N1)
gb|KP097888|_A/duck/Vietnam/NCVD-A672/2011(H5N1)
gb|KF735643|_A/barn_swallow/Hong_Kong/1161/2010(H5N1)
2008
2009
2010
2011
2012
2013
2014
2015
2016

D (NP)

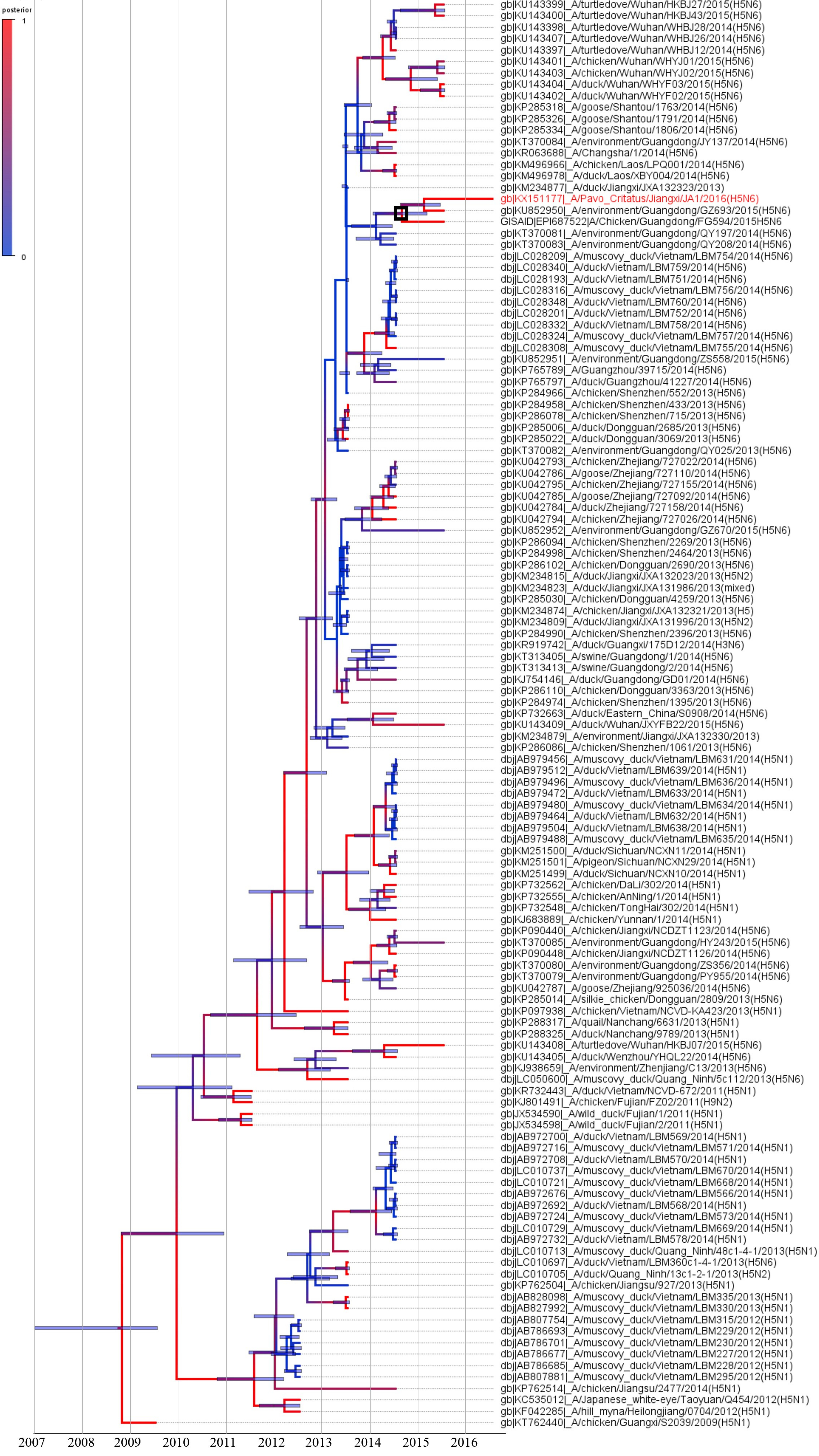
posterior
1
0
gb|KU143399|_A/turtledove/Wuhan/HKBJ27/2015(H5N6)
gb|KU143400|_A/turtledove/Wuhan/HKBJ43/2015(H5N6)
gb|KU143398|_A/turtledove/Wuhan/WHBJ28/2014(H5N6)
gb|KU143407|_A/turtledove/Wuhan/WHBJ26/2014(H5N6)
gb|KU143397|_A/turtledove/Wuhan/WHBJ12/2014(H5N6)
gb|KU143401|_A/chicken/Wuhan/WHYJ01/2015(H5N6)
gb|KU143403|_A/chicken/Wuhan/WHYJ02/2015(H5N6)
gb|KU143404|_A/duck/Wuhan/WHYF03/2015(H5N6)
gb|KU143402|_A/duck/Wuhan/WHYF02/2015(H5N6)
gb|KP285318|_A/goose/Shantou/1763/2014(H5N6)
gb|KP285326|_A/goose/Shantou/1791/2014(H5N6)
gb|KP285334|_A/goose/Shantou/1806/2014(H5N6)
gb|KT370084|_A/environment/Guangdong/JY137/2014(H5N6)
gb|KR063688|_A/Changsha/1/2014(H5N6)
gb|KM496966|_A/chicken/Laos/LPQ001/2014(H5N6)
gb|KM496978|_A/duck/Laos/XBY004/2014(H5N6)
gb|KM234877|_A/duck/Jiangxi/JXA132323/2013)
gb|KX151177|_A/Pavo_Critatus/Jiangxi/JA1/2016(H5N6)
gb|KU852950|_A/environment/Guangdong/GZ693/2015(H5N6)
GISAID|EPI687522|A/Chicken/Guangdong/FG594/2015H5N6
gb|KT370081|_A/environment/Guangdong/QY197/2014(H5N6)
gb|KT370083|_A/environment/Guangdong/QY208/2014(H5N6)
dbj|LC028209|_A/muscovy_duck/Vietnam/LBM754/2014(H5N6)
dbj|LC028340|_A/duck/Vietnam/LBM759/2014(H5N6)
dbj|LC028193|_A/duck/Vietnam/LBM751/2014(H5N6)
dbj|LC028316|_A/muscovy_duck/Vietnam/LBM756/2014(H5N6)
dbj|LC028348|_A/duck/Vietnam/LBM760/2014(H5N6)
dbj|LC028201|_A/duck/Vietnam/LBM752/2014(H5N6)
dbj|LC028332|_A/duck/Vietnam/LBM758/2014(H5N6)
dbj|LC028324|_A/muscovy_duck/Vietnam/LBM757/2014(H5N6)
dbj|LC028308|_A/muscovy_duck/Vietnam/LBM755/2014(H5N6)
gb|KU852951|_A/environment/Guangdong/ZS558/2015(H5N6)
gb|KP765789|_A/Guangzhou/39715/2014(H5N6)
gb|KP765797|_A/duck/Guangzhou/41227/2014(H5N6)
gb|KP284966|_A/chicken/Shenzhen/552/2013(H5N6)
gb|KP284958|_A/chicken/Shenzhen/433/2013(H5N6)
gb|KP286078|_A/chicken/Shenzhen/715/2013(H5N6)
gb|KP285006|_A/duck/Dongguan/2685/2013(H5N6)
gb|KP285022|_A/duck/Dongguan/3069/2013(H5N6)
gb|KT370082|_A/environment/Guangdong/QY025/2013(H5N6)
gb|KU042793|_A/chicken/Zhejiang/727022/2014(H5N6)
gb|KU042786|_A/goose/Zhejiang/727110/2014(H5N6)
gb|KU042795|_A/chicken/Zhejiang/727155/2014(H5N6)
gb|KU042785|_A/goose/Zhejiang/727092/2014(H5N6)
gb|KU042784|_A/duck/Zhejiang/727158/2014(H5N6)
gb|KU042794|_A/chicken/Zhejiang/727026/2014(H5N6)
gb|KU852952|_A/environment/Guangdong/GZ670/2015(H5N6)
gb|KP286094|_A/chicken/Shenzhen/2269/2013(H5N6)
gb|KP284998|_A/chicken/Shenzhen/2464/2013(H5N6)
gb|KP286102|_A/chicken/Dongguan/2690/2013(H5N6)
gb|KM234815|_A/duck/Jiangxi/JXA132023/2013(H5N2)
gb|KM234823|_A/duck/Jiangxi/JXA131986/2013(mixed)
gb|KP285030|_A/chicken/Dongguan/4259/2013(H5N6)
gb|KM234874|_A/chicken/Jiangxi/JXA132321/2013(H5)
gb|KM234809|_A/duck/Jiangxi/JXA131996/2013(H5N2)
gb|KP284990|_A/chicken/Shenzhen/2396/2013(H5N6)
gb|KR919742|_A/duck/Guangxi/175D12/2014(H3N6)
gb|KT313405|_A/swine/Guangdong/1/2014(H5N6)
gb|KT313413|_A/swine/Guangdong/2/2014(H5N6)
gb|KJ754146|_A/duck/Guangdong/GD01/2014(H5N6)
gb|KP286110|_A/chicken/Dongguan/3363/2013(H5N6)
gb|KP284974|_A/chicken/Shenzhen/1395/2013(H5N6)
gb|KP732663|_A/duck/Eastern_China/S0908/2014(H5N6)
gb|KU143409|_A/duck/Wuhan/JXYFB22/2015(H5N6)
gb|KM234879|_A/environment/Jiangxi/JXA132330/2013)
gb|KP286086|_A/chicken/Shenzhen/1061/2013(H5N6)
dbj|AB979456|_A/muscovy_duck/Vietnam/LBM631/2014(H5N1)
dbj|AB979512|_A/duck/Vietnam/LBM639/2014(H5N1)
dbj|AB979496|_A/muscovy_duck/Vietnam/LBM636/2014(H5N1)
dbj|AB979472|_A/duck/Vietnam/LBM633/2014(H5N1)
dbj|AB979480|_A/muscovy_duck/Vietnam/LBM634/2014(H5N1)
dbj|AB979464|_A/duck/Vietnam/LBM632/2014(H5N1)
dbj|AB979504|_A/duck/Vietnam/LBM638/2014(H5N1)
dbj|AB979488|_A/muscovy_duck/Vietnam/LBM635/2014(H5N1)
gb|KM251500|_A/duck/Sichuan/NCXN11/2014(H5N1)
gb|KM251501|_A/pigeon/Sichuan/NCXN29/2014(H5N1)
gb|KM251499|_A/duck/Sichuan/NCXN10/2014(H5N1)
gb|KP732562|_A/chicken/DaLi/302/2014(H5N1)
gb|KP732555|_A/chicken/AnNing/1/2014(H5N1)
gb|KP732548|_A/chicken/TongHai/302/2014(H5N1)
gb|KJ683889|_A/chicken/Yunnan/1/2014(H5N1)
gb|KP090440|_A/chicken/Jiangxi/NCDZT1123/2014(H5N6)
gb|KT370085|_A/environment/Guangdong/HY243/2015(H5N6)
gb|KP090448|_A/chicken/Jiangxi/NCDZT1126/2014(H5N6)
gb|KT370080|_A/environment/Guangdong/ZS356/2014(H5N6)
gb|KT370079|_A/environment/Guangdong/PY955/2014(H5N6)
gb|KU042787|_A/goose/Zhejiang/925036/2014(H5N6)
gb|KP285014|_A/silkie_chicken/Dongguan/2809/2013(H5N6)
gb|KP097938|_A/chicken/Vietnam/NCVD-KA423/2013(H5N1)
gb|KP288317|_A/quail/Nanchang/6631/2013(H5N1)
gb|KP288325|_A/duck/Nanchang/9789/2013(H5N1)
gb|KU143408|_A/turtledove/Wuhan/HKBJ07/2015(H5N6)
gb|KU143405|_A/duck/Wenzhou/YHQL22/2014(H5N6)
gb|KJ938659|_A/environment/Zhenjiang/C13/2013(H5N6)
dbj|LC050600|_A/muscovy_duck/Quang_Ninh/5c112/2013(H5N6)
gb|KR732443|_A/duck/Vietnam/NCVD-672/2011(H5N1)
gb|KJ801491|_A/chicken/Fujian/FZ02/2011(H9N2)
gb|JX534590|_A/wild_duck/Fujian/1/2011(H5N1)
gb|JX534598|_A/wild_duck/Fujian/2/2011(H5N1)
dbj|AB972700|_A/duck/Vietnam/LBM569/2014(H5N1)
dbj|AB972716|_A/muscovy_duck/Vietnam/LBM571/2014(H5N1)
dbj|AB972708|_A/duck/Vietnam/LBM570/2014(H5N1)
dbj|LC010737|_A/muscovy_duck/Vietnam/LBM670/2014(H5N1)
dbj|LC010721|_A/muscovy_duck/Vietnam/LBM668/2014(H5N1)
dbj|AB972676|_A/muscovy_duck/Vietnam/LBM566/2014(H5N1)
dbj|AB972692|_A/duck/Vietnam/LBM568/2014(H5N1)
dbj|AB972724|_A/muscovy_duck/Vietnam/LBM573/2014(H5N1)
dbj|LC010729|_A/muscovy_duck/Vietnam/LBM669/2014(H5N1)
dbj|AB972732|_A/duck/Vietnam/LBM578/2014(H5N1)
dbj|LC010713|_A/muscovy_duck/Quang_Ninh/48c1-4-1/2013(H5N1)
dbj|LC010697|_A/duck/Vietnam/LBM360c1-4-1/2013(H5N6)
dbj|LC010705|_A/duck/Quang_Ninh/13c1-2-1/2013(H5N2)
gb|KP762504|_A/chicken/Jiangsu/927/2013(H5N1)
dbj|AB828098|_A/muscovy_duck/Vietnam/LBM335/2013(H5N1)
dbj|AB827992|_A/muscovy_duck/Vietnam/LBM330/2013(H5N1)
dbj|AB807754|_A/muscovy_duck/Vietnam/LBM315/2012(H5N1)
dbj|AB786693|_A/muscovy_duck/Vietnam/LBM229/2012(H5N1)
dbj|AB786701|_A/muscovy_duck/Vietnam/LBM230/2012(H5N1)
dbj|AB786677|_A/muscovy_duck/Vietnam/LBM227/2012(H5N1)
dbj|AB786685|_A/muscovy_duck/Vietnam/LBM228/2012(H5N1)
dbj|AB807881|_A/muscovy_duck/Vietnam/LBM295/2012(H5N1)
gb|KP762514|_A/chicken/Jiangsu/2477/2014(H5N1)
gb|KC535012|_A/Japanese_white-eye/Taoyuan/Q454/2012(H5N1)
gb|KF042285|_A/hill_myna/Heilongjiang/0704/2012(H5N1)
gb|KT762440|_A/chicken/Guangxi/S2039/2009(H5N1)
2007
2008
2009
2010
2011
2012
2013
2014
2015
2016

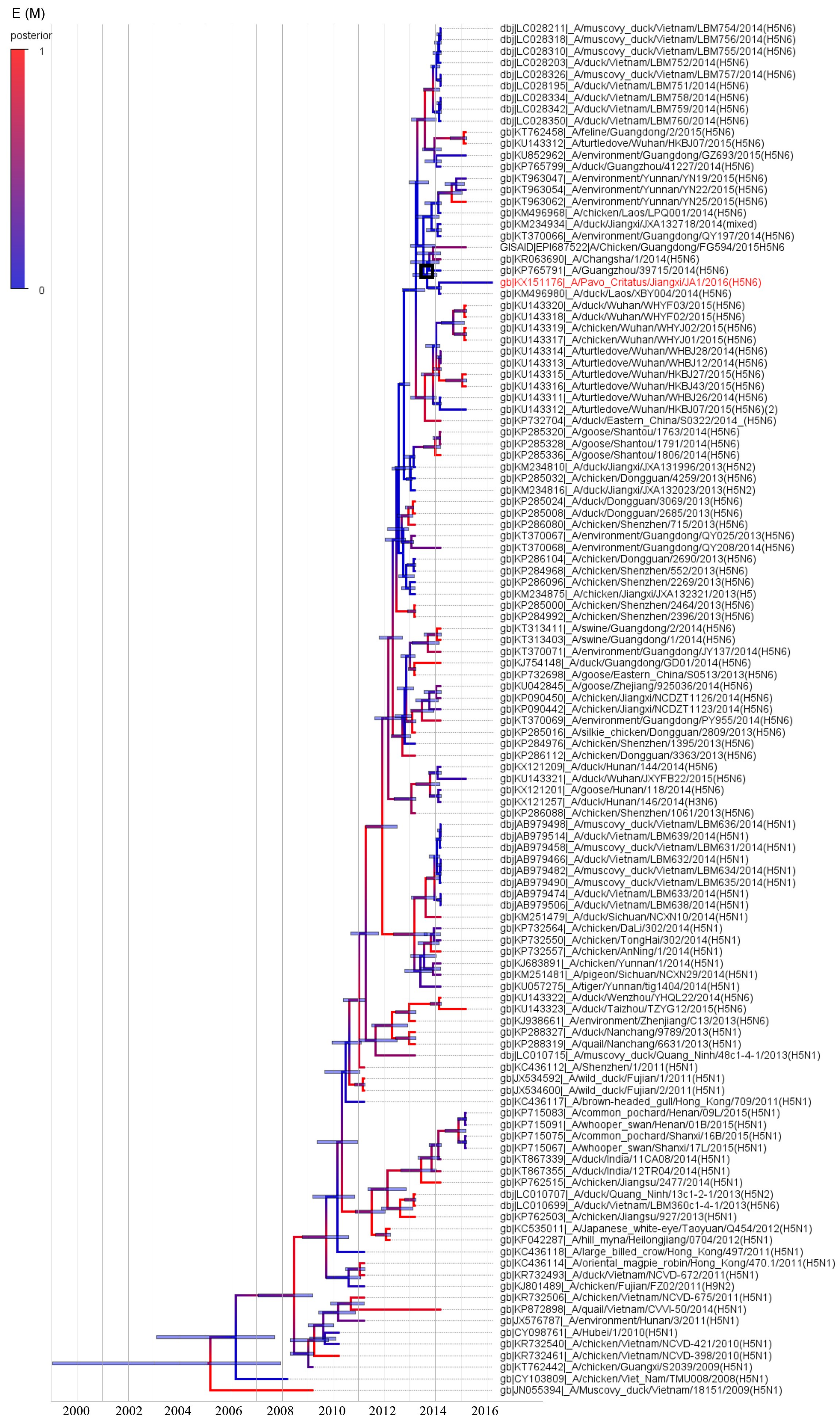
E (M)
posterior
1
0
dbj|LC028211|_A/muscovy_duck/Vietnam/LBM754/2014(H5N6)
dbj|LC028318|_A/muscovy_duck/Vietnam/LBM756/2014(H5N6)
dbj|LC028310|_A/muscovy_duck/Vietnam/LBM755/2014(H5N6)
dbj|LC028203|_A/duck/Vietnam/LBM752/2014(H5N6)
dbj|LC028326|_A/muscovy_duck/Vietnam/LBM757/2014(H5N6)
dbj|LC028195|_A/duck/Vietnam/LBM751/2014(H5N6)
dbj|LC028334|_A/duck/Vietnam/LBM758/2014(H5N6)
dbj|LC028342|_A/duck/Vietnam/LBM759/2014(H5N6)
dbj|LC028350|_A/duck/Vietnam/LBM760/2014(H5N6)
gb|KT762458|_A/feline/Guangdong/2/2015(H5N6)
gb|KU143312|_A/turtledove/Wuhan/HKBJ07/2015(H5N6)
gb|KU852962|_A/environment/Guangdong/GZ693/2015(H5N6)
gb|KP765799|_A/duck/Guangzhou/41227/2014(H5N6)
gb|KT963047|_A/environment/Yunnan/YN19/2015(H5N6)
gb|KT963054|_A/environment/Yunnan/YN22/2015(H5N6)
gb|KT963062|_A/environment/Yunnan/YN25/2015(H5N6)
gb|KM496968|_A/chicken/Laos/LPQ001/2014(H5N6)
gb|KM234934|_A/duck/Jiangxi/JXA132718/2014(mixed)
gb|KT370066|_A/environment/Guangdong/QY197/2014(H5N6)
GISAID|EPI687522|A/Chicken/Guangdong/FG594/2015H5N6
gb|KR063690|_A/Changsha/1/2014(H5N6)
gb|KP765791|_A/Guangzhou/39715/2014(H5N6)
gb|KX151176|_A/Pavo_Critatus/Jiangxi/JA1/2016(H5N6)
gb|KM496980|_A/duck/Laos/XBY004/2014(H5N6)
gb|KU143320|_A/duck/Wuhan/WHYF03/2015(H5N6)
gb|KU143318|_A/duck/Wuhan/WHYF02/2015(H5N6)
gb|KU143319|_A/chicken/Wuhan/WHYJ02/2015(H5N6)
gb|KU143317|_A/chicken/Wuhan/WHYJ01/2015(H5N6)
gb|KU143314|_A/turtledove/Wuhan/WHBJ28/2014(H5N6)
gb|KU143313|_A/turtledove/Wuhan/WHBJ12/2014(H5N6)
gb|KU143315|_A/turtledove/Wuhan/HKBJ27/2015(H5N6)
gb|KU143316|_A/turtledove/Wuhan/HKBJ43/2015(H5N6)
gb|KU143311|_A/turtledove/Wuhan/WHBJ26/2014(H5N6)
gb|KU143312|_A/turtledove/Wuhan/HKBJ07/2015(H5N6)(2)
dbj|KP732704|_A/duck/Eastern_China/S0322/2014_(H5N6)
gb|KP285320|_A/goose/Shantou/1763/2014(H5N6)
gb|KP285328|_A/goose/Shantou/1791/2014(H5N6)
gb|KP285336|_A/goose/Shantou/1806/2014(H5N6)
gb|KM234810|_A/duck/Jiangxi/JXA131996/2013(H5N2)
gb|KP285032|_A/chicken/Dongguan/4259/2013(H5N6)
gb|KM234816|_A/duck/Jiangxi/JXA132023/2013(H5N2)
gb|KP285024|_A/duck/Dongguan/3069/2013(H5N6)
gb|KP285008|_A/duck/Dongguan/2685/2013(H5N6)
gb|KP286080|_A/chicken/Shenzhen/715/2013(H5N6)
gb|KT370067|_A/environment/Guangdong/QY025/2013(H5N6)
gb|KT370068|_A/environment/Guangdong/QY208/2014(H5N6)
gb|KP286104|_A/chicken/Dongguan/2690/2013(H5N6)
gb|KP284968|_A/chicken/Shenzhen/552/2013(H5N6)
gb|KP286096|_A/chicken/Shenzhen/2269/2013(H5N6)
gb|KM234875|_A/chicken/Jiangxi/JXA132321/2013(H5)
gb|KP285000|_A/chicken/Shenzhen/2464/2013(H5N6)
gb|KP284992|_A/chicken/Shenzhen/2396/2013(H5N6)
gb|KT313411|_A/swine/Guangdong/2/2014(H5N6)
gb|KT313403|_A/swine/Guangdong/1/2014(H5N6)
gb|KT370071|_A/environment/Guangdong/JY137/2014(H5N6)
gb|KJ754148|_A/duck/Guangdong/GD01/2014(H5N6)
gb|KP732698|_A/goose/Eastern_China/S0513/2013(H5N6)
gb|KU042845|_A/goose/Zhejiang/925036/2014(H5N6)
gb|KP090450|_A/chicken/Jiangxi/NCDZT1126/2014(H5N6)
gb|KP090442|_A/chicken/Jiangxi/NCDZT1123/2014(H5N6)
gb|KT370069|_A/environment/Guangdong/PY955/2014(H5N6)
gb|KP285016|_A/silkie_chicken/Dongguan/2809/2013(H5N6)
gb|KP284976|_A/chicken/Shenzhen/1395/2013(H5N6)
gb|KP286112|_A/chicken/Dongguan/3363/2013(H5N6)
gb|KX121209|_A/duck/Hunan/144/2014(H5N6)
gb|KU143321|_A/duck/Wuhan/JXYFB22/2015(H5N6)
gb|KX121201|_A/goose/Hunan/118/2014(H5N6)
gb|KX121257|_A/duck/Hunan/146/2014(H3N6)
gb|KP286088|_A/chicken/Shenzhen/1061/2013(H5N6)
dbj|AB979498|_A/muscovy_duck/Vietnam/LBM636/2014(H5N1)
dbj|AB979514|_A/duck/Vietnam/LBM639/2014(H5N1)
dbj|AB979458|_A/muscovy_duck/Vietnam/LBM631/2014(H5N1)
dbj|AB979466|_A/duck/Vietnam/LBM632/2014(H5N1)
dbj|AB979482|_A/muscovy_duck/Vietnam/LBM634/2014(H5N1)
dbj|AB979490|_A/muscovy_duck/Vietnam/LBM635/2014(H5N1)
dbj|AB979474|_A/duck/Vietnam/LBM633/2014(H5N1)
dbj|AB979506|_A/duck/Vietnam/LBM638/2014(H5N1)
gb|KM251479|_A/duck/Sichuan/NCXN10/2014(H5N1)
gb|KP732564|_A/chicken/DaLi/302/2014(H5N1)
gb|KP732550|_A/chicken/TongHai/302/2014(H5N1)
gb|KP732557|_A/chicken/AnNing/1/2014(H5N1)
gb|KJ683891|_A/chicken/Yunnan/1/2014(H5N1)
gb|KM251481|_A/pigeon/Sichuan/NCXN29/2014(H5N1)
gb|KU057275|_A/tiger/Yunnan/tig1404/2014(H5N1)
gb|KU143322|_A/duck/Wenzhou/YHQL22/2014(H5N6)
gb|KU143323|_A/duck/Taizhou/TZYG12/2015(H5N6)
gb|KJ938661|_A/environment/Zhenjiang/C13/2013(H5N6)
gb|KP288327|_A/duck/Nanchang/9789/2013(H5N1)
gb|KP288319|_A/quail/Nanchang/6631/2013(H5N1)
dbj|LC010715|_A/muscovy_duck/Quang_Ninh/48c1-4-1/2013(H5N1)
gb|KC436112|_A/Shenzhen/1/2011(H5N1)
gb|JX534592|_A/wild_duck/Fujian/1/2011(H5N1)
gb|JX534600|_A/wild_duck/Fujian/2/2011(H5N1)
gb|KC436117|_A/brown-headed_gull/Hong_Kong/709/2011(H5N1)
gb|KP715083|_A/common_pochard/Henan/09L/2015(H5N1)
gb|KP715091|_A/whooper_swan/Henan/01B/2015(H5N1)
gb|KP715075|_A/common_pochard/Shanxi/16B/2015(H5N1)
gb|KP715067|_A/whooper_swan/Shanxi/17L/2015(H5N1)
gb|KT867339|_A/duck/India/11CA08/2014(H5N1)
gb|KT867355|_A/duck/India/12TR04/2014(H5N1)
gb|KP762515|_A/chicken/Jiangsu/2477/2014(H5N1)
dbj|LC010707|_A/duck/Quang_Ninh/13c1-2-1/2013(H5N2)
dbj|LC010699|_A/duck/Vietnam/LBM360c1-4-1/2013(H5N6)
gb|KP762503|_A/chicken/Jiangsu/927/2013(H5N1)
gb|KC535011|_A/Japanese_white-eye/Taoyuan/Q454/2012(H5N1)
gb|KF042287|_A/hill_myna/Heilongjiang/0704/2012(H5N1)
gb|KC436118|_A/large_billed_crow/Hong_Kong/497/2011(H5N1)
gb|KC436114|_A/oriental_magpie_robin/Hong_Kong/470.1/2011(H5N1)
gb|KR732493|_A/duck/Vietnam/NCVD-672/2011(H5N1)
gb|KJ801489|_A/chicken/Fujian/FZ02/2011(H9N2)
gb|KR732506|_A/chicken/Vietnam/NCVD-675/2011(H5N1)
gb|KP872898|_A/quail/Vietnam/CVVI-50/2014(H5N1)
gb|JX576787|_A/environment/Hunan/3/2011(H5N1)
gb|CY098761|_A/Hubei/1/2010(H5N1)
gb|KR732540|_A/chicken/Vietnam/NCVD-421/2010(H5N1)
gb|KR732461|_A/chicken/Vietnam/NCVD-398/2010(H5N1)
gb|KT762442|_A/chicken/Guangxi/S2039/2009(H5N1)
gb|CY103809|_A/chicken/Viet_Nam/TMU008/2008(H5N1)
gb|JN055394|_A/Muscovy_duck/Vietnam/18151/2009(H5N1)
2000
2002
2004
2006
2008
2010
2012
2014
2016

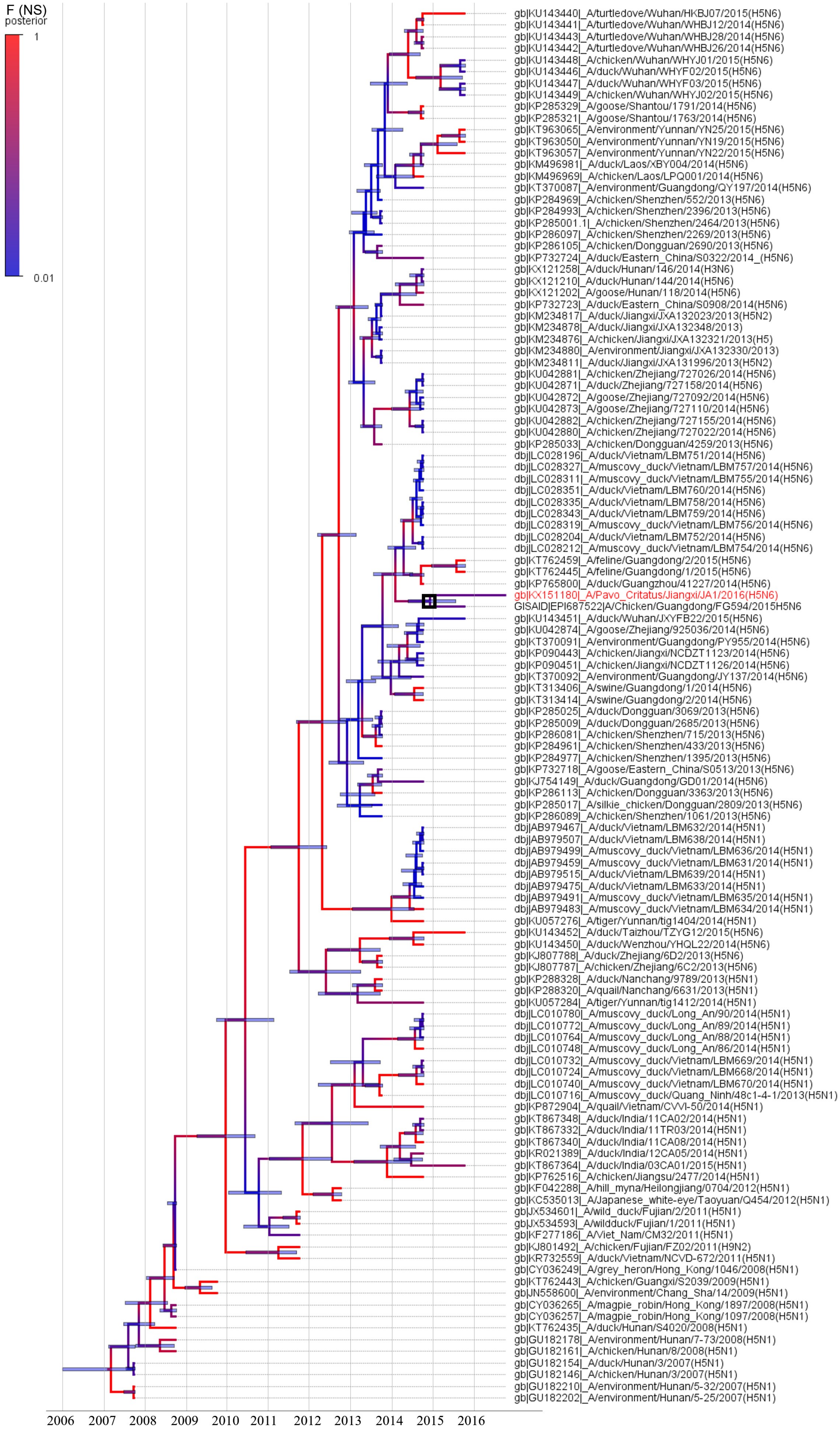
F (NS)
posterior
1
0.01
gb|KU143440|_A/turtledove/Wuhan/HKBJ07/2015(H5N6)
gb|KU143441|_A/turtledove/Wuhan/WHBJ12/2014(H5N6)
gb|KU143443|_A/turtledove/Wuhan/WHBJ28/2014(H5N6)
gb|KU143442|_A/turtledove/Wuhan/WHBJ26/2014(H5N6)
gb|KU143448|_A/chicken/Wuhan/WHYJ01/2015(H5N6)
gb|KU143446|_A/duck/Wuhan/WHYF02/2015(H5N6)
gb|KU143447|_A/duck/Wuhan/WHYF03/2015(H5N6)
gb|KU143449|_A/chicken/Wuhan/WHYJ02/2015(H5N6)
gb|KP285329|_A/goose/Shantou/1791/2014(H5N6)
gb|KP285321|_A/goose/Shantou/1763/2014(H5N6)
gb|KT963065|_A/environment/Yunnan/YN25/2015(H5N6)
gb|KT963050|_A/environment/Yunnan/YN19/2015(H5N6)
gb|KT963057|_A/environment/Yunnan/YN22/2015(H5N6)
gb|KM496981|_A/duck/Laos/XBY004/2014(H5N6)
gb|KM496969|_A/chicken/Laos/LPQ001/2014(H5N6)
gb|KT370087|_A/environment/Guangdong/QY197/2014(H5N6)
gb|KP284969|_A/chicken/Shenzhen/552/2013(H5N6)
gb|KP284993|_A/chicken/Shenzhen/2396/2013(H5N6)
gb|KP285001.1|_A/chicken/Shenzhen/2464/2013(H5N6)
gb|KP286097|_A/chicken/Shenzhen/2269/2013(H5N6)
gb|KP286105|_A/chicken/Dongguan/2690/2013(H5N6)
gb|KP732724|_A/duck/Eastern_China/S0322/2014_(H5N6)
gb|KX121258|_A/duck/Hunan/146/2014(H3N6)
gb|KX121210|_A/duck/Hunan/144/2014(H5N6)
gb|KX121202|_A/goose/Hunan/118/2014(H5N6)
gb|KP732723|_A/duck/Eastern_China/S0908/2014(H5N6)
gb|KM234817|_A/duck/Jiangxi/JXA132023/2013(H5N2)
gb|KM234878|_A/duck/Jiangxi/JXA132348/2013)
gb|KM234876|_A/chicken/Jiangxi/JXA132321/2013(H5)
gb|KM234880|_A/environment/Jiangxi/JXA132330/2013)
gb|KM234811|_A/duck/Jiangxi/JXA131996/2013(H5N2)
gb|KU042881|_A/chicken/Zhejiang/727026/2014(H5N6)
gb|KU042871|_A/duck/Zhejiang/727158/2014(H5N6)
gb|KU042872|_A/goose/Zhejiang/727092/2014(H5N6)
gb|KU042873|_A/goose/Zhejiang/727110/2014(H5N6)
gb|KU042882|_A/chicken/Zhejiang/727155/2014(H5N6)
gb|KU042880|_A/chicken/Zhejiang/727022/2014(H5N6)
gb|KP285033|_A/chicken/Dongguan/4259/2013(H5N6)
dbj|LC028196|_A/duck/Vietnam/LBM751/2014(H5N6)
dbj|LC028327|_A/muscovy_duck/Vietnam/LBM757/2014(H5N6)
dbj|LC028311|_A/muscovy_duck/Vietnam/LBM755/2014(H5N6)
dbj|LC028351|_A/duck/Vietnam/LBM760/2014(H5N6)
dbj|LC028335|_A/duck/Vietnam/LBM758/2014(H5N6)
dbj|LC028343|_A/duck/Vietnam/LBM759/2014(H5N6)
dbj|LC028319|_A/muscovy_duck/Vietnam/LBM756/2014(H5N6)
dbj|LC028204|_A/duck/Vietnam/LBM752/2014(H5N6)
dbj|LC028212|_A/muscovy_duck/Vietnam/LBM754/2014(H5N6)
gb|KT762459|_A/feline/Guangdong/2/2015(H5N6)
gb|KT762445|_A/feline/Guangdong/1/2015(H5N6)
gb|KP765800|_A/duck/Guangzhou/41227/2014(H5N6)
gb|KX151180|_A/Pavo_Critatus/Jiangxi/JA1/2016(H5N6)
GISAID|EPI687522|A/Chicken/Guangdong/FG594/2015H5N6
gb|KU143451|_A/duck/Wuhan/JXYFB22/2015(H5N6)
gb|KU042874|_A/goose/Zhejiang/925036/2014(H5N6)
gb|KT370091|_A/environment/Guangdong/PY955/2014(H5N6)
gb|KP090443|_A/chicken/Jiangxi/NCDZT1123/2014(H5N6)
gb|KP090451|_A/chicken/Jiangxi/NCDZT1126/2014(H5N6)
gb|KT370092|_A/environment/Guangdong/JY137/2014(H5N6)
gb|KT313406|_A/swine/Guangdong/1/2014(H5N6)
gb|KT313414|_A/swine/Guangdong/2/2014(H5N6)
gb|KP285025|_A/duck/Dongguan/3069/2013(H5N6)
gb|KP285009|_A/duck/Dongguan/2685/2013(H5N6)
gb|KP286081|_A/chicken/Shenzhen/715/2013(H5N6)
gb|KP284961|_A/chicken/Shenzhen/433/2013(H5N6)
gb|KP284977|_A/chicken/Shenzhen/1395/2013(H5N6)
gb|KP732718|_A/goose/Eastern_China/S0513/2013(H5N6)
gb|KJ754149|_A/duck/Guangdong/GD01/2014(H5N6)
gb|KP286113|_A/chicken/Dongguan/3363/2013(H5N6)
gb|KP285017|_A/silkie_chicken/Dongguan/2809/2013(H5N6)
gb|KP286089|_A/chicken/Shenzhen/1061/2013(H5N6)
dbj|AB979467|_A/duck/Vietnam/LBM632/2014(H5N1)
dbj|AB979507|_A/duck/Vietnam/LBM638/2014(H5N1)
dbj|AB979499|_A/muscovy_duck/Vietnam/LBM636/2014(H5N1)
dbj|AB979459|_A/muscovy_duck/Vietnam/LBM631/2014(H5N1)
dbj|AB979515|_A/duck/Vietnam/LBM639/2014(H5N1)
dbj|AB979475|_A/duck/Vietnam/LBM633/2014(H5N1)
dbj|AB979491|_A/muscovy_duck/Vietnam/LBM635/2014(H5N1)
dbj|AB979483|_A/muscovy_duck/Vietnam/LBM634/2014(H5N1)
gb|KU057276|_A/tiger/Yunnan/tig1404/2014(H5N1)
gb|KU143452|_A/duck/Taizhou/TZYG12/2015(H5N6)
gb|KU143450|_A/duck/Wenzhou/YHQL22/2014(H5N6)
gb|KJ807788|_A/duck/Zhejiang/6D2/2013(H5N6)
gb|KJ807787|_A/chicken/Zhejiang/6C2/2013(H5N6)
gb|KP288328|_A/duck/Nanchang/9789/2013(H5N1)
gb|KP288320|_A/quail/Nanchang/6631/2013(H5N1)
gb|KU057284|_A/tiger/Yunnan/tig1412/2014(H5N1)
dbj|LC010780|_A/muscovy_duck/Long_An/90/2014(H5N1)
dbj|LC010772|_A/muscovy_duck/Long_An/89/2014(H5N1)
dbj|LC010764|_A/muscovy_duck/Long_An/88/2014(H5N1)
dbj|LC010748|_A/muscovy_duck/Long_An/86/2014(H5N1)
dbj|LC010732|_A/muscovy_duck/Vietnam/LBM669/2014(H5N1)
dbj|LC010724|_A/muscovy_duck/Vietnam/LBM668/2014(H5N1)
dbj|LC010740|_A/muscovy_duck/Vietnam/LBM670/2014(H5N1)
dbj|LC010716|_A/muscovy_duck/Quang_Ninh/48c1-4-1/2013(H5N1)
gb|KP872904|_A/quail/Vietnam/CVVI-50/2014(H5N1)
gb|KT867348|_A/duck/India/11CA02/2014(H5N1)
gb|KT867332|_A/duck/India/11TR03/2014(H5N1)
gb|KT867340|_A/duck/India/11CA08/2014(H5N1)
gb|KR021389|_A/duck/India/12CA05/2014(H5N1)
gb|KT867364|_A/duck/India/03CA01/2015(H5N1)
gb|KP762516|_A/chicken/Jiangsu/2477/2014(H5N1)
gb|KF042288|_A/hill_myna/Heilongjiang/0704/2012(H5N1)
gb|KC535013|_A/Japanese_white-eye/Taoyuan/Q454/2012(H5N1)
gb|JX534601|_A/wild_duck/Fujian/2/2011(H5N1)
gb|JX534593|_A/wildduck/Fujian/1/2011(H5N1)
gb|KF277186|_A/Viet_Nam/CM32/2011(H5N1)
gb|KJ801492|_A/chicken/Fujian/FZ02/2011(H9N2)
gb|KR732559|_A/duck/Vietnam/NCVD-672/2011(H5N1)
gb|CY036249|_A/grey_heron/Hong_Kong/1046/2008(H5N1)
gb|KT762443|_A/chicken/Guangxi/S2039/2009(H5N1)
gb|JN558600|_A/environment/Chang_Sha/14/2009(H5N1)
gb|CY036265|_A/magpie_robin/Hong_Kong/1897/2008(H5N1)
gb|CY036257|_A/magpie_robin/Hong_Kong/1097/2008(H5N1)
gb|KT762435|_A/duck/Hunan/S4020/2008(H5N1)
gb|GU182178|_A/environment/Hunan/7-73/2008(H5N1)
gb|GU182161|_A/chicken/Hunan/8/2008(H5N1)
gb|GU182154|_A/duck/Hunan/3/2007(H5N1)
gb|GU182146|_A/chicken/Hunan/3/2007(H5N1)
gb|GU182210|_A/environment/Hunan/5-32/2007(H5N1)
gb|GU182202|_A/environment/Hunan/5-25/2007(H5N1)
2006
2007
2008
2009
2010
2011
2012
2013
2014
2015
2016

Supplementary Figure 2. Genesis analysis of JA1/2016. The phylogenetic trees were conducted using gene sequences listed in the NCBI Blast analysis. Panels (A) to (E) represent PB2, PB1, PA, NP, M, and NS separately. The black square on the internal nodes means the divergent nodes of JA1/2016 and FG594-like virus. All the trees were built by BEAST (v 1.8.4) and displayed using FigTree (v 1.4.2).

Supplementary Figure 3. Estimation of effective population size of HA and NA genes of these H5N6 viruses using the Bayesian SkyGrid model. The left axis represents the effective population dynamics of HA or NA genes, and black line and shaded blue region represent the median and 95% high posterior density (HPD) intervals of HA and NA genes of H5N6 viruses.

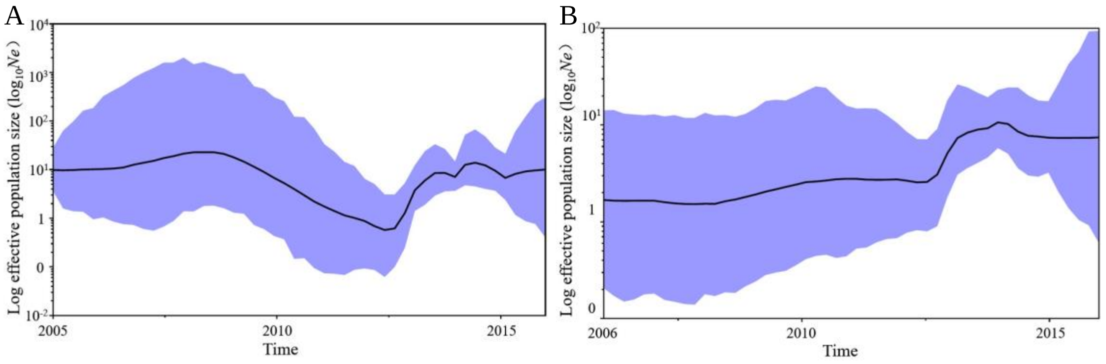

Supplementary Table S1. Dataset size, and best-fit model for each gene segment.

Gene segment datasets	Sequence Numbers	Substitution Models	Relax-clock models[a]	Tree Models	MCMC chain length[b]
PB2	112	GTR+G+I	uced	Bayesian SkyGrid	50
PB1	121	GTR+G	uced	Bayesian SkyGrid	50
PA	120	GTR+G	uced	Exponential Growth	50
HA	111	GTR+G	uced	Exponential Growth	50
NP	125	GTR+I	uced	Exponential Growth	50
NA	102	HKY+G	uced	Exponential Growth	50
M	119	HKY+G	uced	Bayesian SkyGrid	50
NS	120	GTR+G	uced	Bayesian SkyGrid	50
HA (2012-2016)	580	GTR+G+I	uced	Exponential Growth	500
NA (2012-2016)	448	GTR+G+I	uced	Exponential Growth	500

[a] uced: uncorrelated exponential clock model

[b] the unit was million

Supplementary Table S2: The best-fit models were estimated by Model Comparison (AICM method with 1000 bootstraps) implemented in Tracer (v1.6). Lower AICM values indicate better model fit. The S.E. are shown in the square brackets.

Genes	ucld-Constant	ucld-Exponential	ucld-SkyGrid
PB2	22,360.486[±0.438]	22,365.951[±0.558]	22,360.366[±0.703]
PB1	14,723.275[±0.415]	14,709.917[±0.986]	14,697.933[±0.784]
PA	12,896.398[±0.358]	12,933.927[±0.659]	12,923.57[±0.485]
HA	13,922.524[±0.868]	13,918.21[±0.913]	13,946.507[±0.816]
NP	9,177.478[±0.312]	9,175.421[±0.472]	9,176.24[±0.834]
NA	10,392.331[±0.244]	10,398.492[±0.493]	10,389.199[±0.477]
M	6,058.971[±0.395]	6,051.881[±0.951]	6,044.605[±0.588]
NS	5,507.486[±0.405]	5,520.918[±0.448]	5,501.45[±0.405]
HA(2012-2016)	68,419.589[4.693]	66,823.114[6.328]	68,655.714[8.161]
NA(2012-2016)	45,188.308[4.209]	45,206.582[7.181]	47,432.777[9.923]

Genes	ucgd-Constant	ucgd-Exponential	ucgd-SkyGrid
PB2	22,347.39[±0.559]	22,348.471[±0.739]	22,347.139[±0.659]
PB1	14,692.683[±0.966]	14,693.177[±0.602]	14,716.158[±0.848]
PA	12,876.684[±0.396]	12,878.995[±0.52]	12,872.285[±0.336]
HA	13,897.093[±0.387]	13,903.651[±0.341]	13,910.445[±0.655]
NP	9,150.322[±0.677]	9,143.621[±0.845]	9,146.387[±0.283]
NA	10,372.528[±0.779]	10,381.585[±0.553]	10,376.747[±0.498]
M	6,018.114[±0.588]	6,020.398[±0.599]	6,010.274[±0.481]
NS	5,480.133[±0.539]	5,472.664[±0.372]	5,469.348[±0.604]
HA (2012-2016)	67,019.333[11.259]	66,572.663[4.241]	67,813.712[9.555]
NA (2012-2016)	46,087.49[4.132]	45,491.416[6.132]	45,183.808[4.821]

Genes	uced-Constant	uced-Exponential	uced-SkyGrid
PB2	22,314.897[±0.438]	22,365.951[±0.658]	**22,311.331[±0.449]**
PB1	14,688.446[±0.387]	14,688.56[±0.368]	**14,672.143[±0.875]**
PA	12,872.424[±0.555]	**12,855.375[±0.65]**	12,873.784[±0.691]
HA	13,896.623[±0.337]	**13,880.867[±0.866]**	13,899.74[±0.472]
NP	9,133.123[±0.525]	**9,126.031[±0.56]**	9,136.632[±0.318]
NA	10,353.247[±0.428]	**10,341.072[±0.584]**	10,348.811[±0.814]
M	6,006.754[±0.481]	6,009.721[±0.914]	**6,001.153[±0.914]**
NS	5,471.09[±0.517]	5,472.579[±0.791]	**5,457.65[±0.443]**
HA (2012-2016)	67992.397[5.151]	**65,363.595[12.838]**	65,463.874[8.464]
NA (2012-2016)	44,984.981[8.925]	**44,948.851[6.231]**	45,373.474[5.726]

ucld: uncorrelated log-normal relax-clock model; ucgd: uncorrelated gammar relax-clock model; uced: uncorrelated exponential relax-clock model.

Constant: coalescent Constant Size; Exponential: coalescent Exponential Growth; SkyGrid: coalescent Bayesian SkyGrid

Supplementary Tables 3. Molecular characteristics of JA1/2016 were analyzed using Influenza Research Datebase (IRD, https://www.fludb.org/)

Protein	Molecular Feature	Function
PB2	I63	The mutations I63T in PB2 and T677M in PB1 co-mediate the reduced pathogenicity of H5N1 viruses.
PB2	V89, D309, K339, G477, V495, E627,	Introduction of L89V, G309D, T339K, R477G, I495V, K627E, A676T in the A/wild duck/Hunan/021/2005 backbone conferred increased polymerase activity in mouse cells.
PB2	A199	The presence of an A199S substitution enhances viral transmission to humans.
PB2	D256	The D256G and E627K amino acid substitutions play a role in the adaptation of influenza viruses to mammalian cells.
PB2	T271	The T271A mutation showed enhanced virus growth in mammalian cells in vitro.
PB2	V588	The amino acid residues V588, L636 and D701 act as host range-determinants and together compensate for E627.
PB2	Q591	Substitution of Q591K conferred increased replication and virulence in mamallian cells.
PB2	L636	The L636F improves virus activity in mammalian cells.
PB2	E627	E627K contributes to viral transmission in mammal ainimals, and conferred increased airborne transmission in ferrets.
PB2	S661	The presence of an A661T substitution enhances viral transmission to humans.
PB2	V667	The presence of an V667I substitution enhances viral transmission to humans.
PB2	D701	D701N contributes to viral transmission in mammal ainimals; and conferred increased airborne transmission in ferrets.
PB2	K702	The presence of an K702R substitution enhances viral transmission to humans.
PB2	S714	S714R confers adaptation of avian influenza virus to the mammalian host.
PB1	V3, N328, N375	Introduction of V3A, N328K, and N375S substitutions in the A/Vietnam/1203/2004 backbone conferred increased virulence in mice.
PB1	P13, S678	The PB1 P13 and N678, together with PB2 701N and 714R, PA 615N, and NP 319K cause a dramatic increase in polymerase activity and confer adaptation of avian influenza virus to the mammalian host.
PB1	H99, I368	Introduction of H99Y and I368V in the A/Indonesia/5/2005 backbone conferred increased airborne transmission in mammals.
PB1	K207	Introduction of K207R in the A/Vietnam/1203/2004 backbone conferred increased virulence and decreased polymerase activity.
PB1	Y436	The Y436H mutation in PB1 compromises transmissibility as viruses with this change are not transmitted efficiently in ducks.
PB1	V473, L598	Introduction of V473L and P598L substitutions in recombinant virus conferred decreased polymerase activity in 293 Tcells.
PB1-F2	Truncated PB1-F2	Unclear
PA	T97	The T97I in PA causes enhanced virulence and replication in mice due to enhanced polymerase activity.
PA	S409	The presence of S409N enhances transmission in humans.
PA	T515	Introduction of T515A substitutions in the A/Vietnam/1203/2004 backbone conferred decreased polymerase activity as indicated by the luciferase activity, caused no mortality in ducks.
PA	T85, G186, L336	These residues are responsible for the enhanced polymerase activity in mammalian cells.
PA	L672	Present of L672 contributes to airborne transmission of influenza virus.
HA	Y7	Y23H has been shown to result in an increase in the pH of HA conformational change and membrane fusion.
HA	H8	H24Q has been shown to decrease the pH of HA conformational change and membrane fusion.
HA	N94	Introduction of A94N substitution in the A/chicken/Fujian/1042/05 backbone conferred increased binding to alpha 2-6 receptor.

HA	S121	Introduction of S121N in the A/Vietnam/1203/2004 backbone conferred increased binding to alpha 2-6 by measuring hemagglutination activities using enzymatically modified chicken RBCs.
HA	A133	Introduction of S133A substitution in the A/Thailand/KAN 1/2004 backbone conferred alpha 2-6 linked receptor binding capacity.
HA	A134	Residue 134 of HA alters the receptor-binding property of the virus.
HA	(-)129, A134	The substitutions L145V and A150V can change the receptor-binding preference of HA of H5N1 virus from SAalpha2,3Gal to both SAalpha2,3Gal and the human-type SAalpha2,6Gal receptor.
HA	N154	The loss of a potential glycosylation site at residue 154 of the HA enhanced the virulence of the H5N1 virus for mice.
HA	D155	Introduction of S155N naturally occurring substitution in the A/Vietnam/1203/2004 backbone conferred increased binding to alpha2-6 without loss of binding to alpha2-3 by comparing HA activities using enzymatically modified chicken RBCs.
HA	A156	Introduction of T156A in the A/Vietnam/1203/2004 backbone conferred increased binding to alpha 2-6.
HA	N166	Introduction of N166K in the A/Indonesia/5/2005(H5N1) backbone conferred increased binding to alpha 2-6 using a solid phase binding assay with the sodium salts of sialylglycopoymers.
HA	N183	Introduction of D183G substitution in the A/Vietnam/1203/2004 backbone conferred increased binding to alpha 2-6.
HA	E186	Introduction of E186G substitution in the A/Vietnam/1203/2004 backbone conferred increased binding to alpha2-6.
HA	T188	Introduction of T188I substitution in the A/Thailand/KAN 1/2004 backbone conferred alpha 2-6 linked receptor binding suing glycan microarrays.
HA	N189	Unclear, however, introduction of K189R in the A/Vietnam/1203/2004 backbone conferred increased binding to alpha 2-6 without loss of binding to alpha 2-3 by comparing HA activities using enzymatically modified chicken RBCs.
HA	K192	Unclear, however, the Q192R mutation in the HA of VN1194 (H5N1) virus significantly enhanced the capacity of the HA to recognize SAa2,6Gal causing a the shift to recognition of the human receptor.
HA	N193	Introduction of N193K substitution in the A/Vietnam/1194/2004xPR8 backbone conferred increased binding to alpha 2-6 using a solid phase binding assay with the sodium salts of sialylglycopoymers.
HA	V210	Introduction of V226I substitution in the A/duck/Egypt/D1Br12/2007 backbone conferred increased binding to alpha 2-6 using solid phase direct binding assay with sialyglycopolymer containing N-acetylneuraminic acid linked to galactose.
HA	Q218	Unclear, but introduction of K218E substitution in the A/Thailand/KAN 1/2004 backbone conferred increased replication efficiency since the virus replicated to high titers at each time point investigated in lung.The mutant also decreased virulence as indicated by lethal dose in mice.
HA	Q222	Introduction of Gln238Leu substitution in the A/Indonesia/05/2005 backbone conferred agglutinated alpha 2-6 but not alpha 2-3 in turkey red blood cells (TRBC) using hemagglutination assay.
HA	R223	Unclear, Introduction of S223N substitution in the A/Vietnam/1203/2004 backbone conferred increased binding to 6' sialyl lactosamine relative to WT parental virus using ELISA based assay.
HA	G224	Introduction of G224S substitution in virus conferred increased binding to SAalpha 2-6.
HA	P235	Introduction of S235P substitution in the A/duck/Egypt/D1Br12/2007 backbone conferred slight increased binding to alpha 2-6 using solid phase binding assay.
HA	E251	Introduction of E251K substitution in the A/Vietnam/1203/2004 backbone increased binding to 6' sialyl lactosamine relative to WT parental virus using ELISA based assay.
HA	K388	Mutation at this site to I has been shown to decrease the pH of HA conformational change and membrane fusion.

HA	E435	Mutation at this site to K has been shown to decrease the pH of HA conformational change and membrane fusion.
HA	D442	Mutation at this site to G or N has been shown to increase the pH of HA conformational change, and when substituted with N also results in an increase in the pH of membrane fusion.
HA	N444	Mutation at this site to K has been shown to increase the pH of HA conformational change and membrane fusion.
HA	H103, A156, Q222, G224	Introduction of the His103Tyr, Thr156Ala, Gln222Leu, Gly224Ser naturally occurring substitutions in the A/Indonesia/5/2005 backbone conferred increased airborne transmission in ferrets using paired transmission cages.
HA	E75,N193	Introduction of Ser134Pro, Asn204Lys substitutions in the A/Vietnam/1194/2004 (HA,NA) x PR8 backbone conferred increased binding to alpha 2-6 using a solid phase binding assay with the sodium salts of sialylglycopoymers.
HA	E75, R497	
HA	A149,T204	Introduction of Ser149Ala, Thr204Ile substitutions in the A/Thailand/KAN 1/2004 backbone conferred alpha 2-6 linked receptor binding using resialylated hemagglutination assay.
HA	H103,A156,Q222,G224	Introduction of the His103Tyr, Thr156Ala, Gln222Leu, Gly224Ser naturally occurring substitutions in the A/Indonesia/5/2005 backbone conferred increased airborne transmission in ferrets using paired transmission cages.
HA	R323, E324, R325, R326, R327, K328, -329, R330	This motif RERRRKR is responsible for highly pathogenic of influenza virus
NP	I105	Valine at position 105 in NP is critical for the high pathogenicity.
NP	L136	L136M enhances transmission to humans
NP	K184	A change from A to K at residue 184 of NP results in increased replication and pathogenicity of the viruses in chickens.
NP	R99,S345	Introduction of R99K and S345N in the A/Indonesia/5/2005 backbone conferred airborne transmission in mammals.
NP	N319	
M1	D30,A215	Introduction of Asn30Asp and Thr215Ala substitutions in the A/duck/Guangxi/53/2002 backbone conferred increased virulence in mice indicated by survival rate.
M1	D30,V142,N207,A209	Single replacement of either one of the residue in M1 protein reduces overall viral production as well as growth kinetics.
M1	T139	The mouse adapted A/Fort Monmouth/1/47 virus contained Thr139Ala substitution that conferred increased virulence as indicated by measuring median lethal dose in mice and increased viral yield in lungs of mice and MDCK cells.
M2	E16	A16G substitution leads to enhanced transmission in humans
M2	L26	26F confers resistance to Adamantane
M2	V27	27A confers resistance to Adamantane
M2	A30	30T confers resistance to Adamantane
M2	S31	31N confers resistance to Adamantane
M2	G34	34E confers resistance to Adamantane
M2	Y50	The viruses lacking the palmitoylation site at this residue have been shown to cause a modest reduction in virulence in vivo (mouse models) although the effect is not seen tissue culture cells.
M2	L55	C55F substitution leads to enhanced transmission in humans

NS1	S42	Introduction of P42S substitution conferred increased virulence as indicated by lethality in mice and the systemic spread of infection. This substitution also affects IFN pathway.
NS1	Y89	This residue is present in the highly conserved src homology (SH)–binding motifs within NS1 and the point mutation Y89F results in restricted virus spread in mouse lung and reduced virulence phenotype.
NS1	E92	This residue of NS1 is responsible for H5N1 viruses' resistance to the interferons and TNF-alpha and depends on the substitution of Glu for aspartic acid at position 92 of the NS1 molecule.
NS1	F103,M106	Either L103F or I106M in the HK97 NS1A protein caused increase in the rate of virus replication. HK97 NS1A protein is intrinsically defective in binding CPSF30.
NS1	D125	The single mutation D125G causes high pathogenicity in mice and also causes enhanced binding abilities to a2,3 and a2,6 sialic acid-linked
NS1	A149	Influenza virus with A149 can antagonize the induction of interferon levels in chicken embryo fibroblasts (CEFs), but a recombinants with V149 are not capable of the same effect.
NS1	L90, T91, E92, M93, T94, L95, E96, E97, M98, S99	The changed length of the NS1 eIF4GI binding domain in H5N1 viruses with a 5-amino acid deletion can cause increased virulence and pathogenicity. Truncation of the eIF4GI binding domain attenuates replication invitro and invivo.
NS1	S205, G210	Residues at positions 200 and 205 of NS1 contribute to enhanced type I interferon (IFN) antagonistic activity.
NS1	E227,S228,E229,V230	The avian and equine strains always have ESEV at the C-terminal region and human almost never have that. In contrast, majority of the human signatures have RSKV sequence; the remaining have avian signatures and are known to be of avian origin.
NS1	-80,-81,-82,-83,-84	Introduction of an artificial 15nt deletion in the recombinant virus conferred increased the virulence in mice using lethal dose and using intravenous pathogenicity index in chickens.
NS1	I226,E227,S228,E229,V230	Introduction of the PL motif at the C terminal in the virus A/WSN/33 conferred significant weight loss compared to WT. The virus variant showed severe alveolitis and hemorrhage in lung tissue of mice.
NS2	E47,R51	Residues 47 and 51 of NS2 are associated with difference in virulence between high and low pathogenic H5N1 viruses in ferrets.
